# Somatic Tumor Next-Generation Sequencing in US Veterans With Metastatic Prostate Cancer

**DOI:** 10.1001/jamanetworkopen.2025.9119

**Published:** 2025-05-12

**Authors:** Luca F. Valle, Jiannong Li, Heena Desai, Ryan Hausler, Candace Haroldsen, Monica Chatwal, Matthias Ojo, Michael J. Kelley, Timothy R. Rebbeck, Brent S. Rose, Matthew B. Rettig, Nicholas G. Nickols, Isla P. Garraway, Kosj Yamoah, Kara N. Maxwell

**Affiliations:** 1 Radiation Oncology Service, Veterans Affairs (VA) Greater Los Angeles Healthcare System, Los Angeles, California; 2 Department of Radiation Oncology, UCLA (University of California, Los Angeles), Los Angeles; 3 UCLA Jonsson Comprehensive Cancer Center, Los Angeles, California; 4 Department of Biostatistics and Bioinformatics, H. Lee Moffitt Cancer Center, Tampa, Florida; 5 Medical Oncology Service, Corporal Michael Crescenz VA Medical Center, Philadelphia, Pennsylvania; 6 Division of Hematology-Oncology, Department of Medicine, Perelman School of Medicine, University of Pennsylvania, Philadelphia; 7 Division of Epidemiology, Department of Internal Medicine, University of Utah, Salt Lake City; 8 VA Salt Lake City Healthcare System, Salt Lake City, Utah; 9 Department of Genitourinary Oncology, H. Lee Moffitt Cancer Center, Tampa, Florida; 10 Hematology-Oncology Service, James Haley VA Hospital, Tampa, Florida; 11 Charles R. Drew University School of Medicine and Science, Los Angeles, California; 12 National Oncology Program, Department of Veterans Affairs, Washington, DC; 13 Hematology-Oncology Service, Durham VA Medical Center, Durham, North Carolina; 14 Department of Medicine and Duke Cancer Institute, Duke University, Durham, North Carolina; 15 Harvard T. H. Chan School of Public Health and Dana-Farber Cancer Institute, Boston, Massachusetts; 16 Department of Radiation Medicine and Applied Sciences, University of California, San Diego, School of Medicine, La Jolla; 17 Veterans Health Administration San Diego Health Care System, La Jolla, California; 18 Department of Medicine, VA Greater Los Angeles Healthcare System, Los Angeles, California; 19 Department of Medicine, David Geffen School of Medicine at UCLA, Los Angeles; 20 Department of Urology, David Geffen School of Medicine at UCLA, Los Angeles; 21 Department of Surgical and Perioperative Care, VA Greater Los Angeles Healthcare System, Los Angeles, California; 22 Radiation Oncology Service, James Haley VA Medical Center, Tampa, Florida; 23 Department of Radiation Oncology, H. Lee Moffitt Cancer Center, Tampa, Florida; 24 Abramson Cancer Center, Perelman School of Medicine, University of Pennsylvania, Philadelphia

## Abstract

**Importance:**

National guidelines recommend next-generation sequencing (NGS) of tumors in patients diagnosed with metastatic prostate cancer (mPCa) to identify potential actionable alterations. Non-Hispanic Black men are poorly represented in precision oncology cohorts, and therefore differences in alterations frequencies between non-Hispanic Black and White men remain poorly characterized.

**Objectives:**

To describe the spectrum and frequency of alterations in PCa-related genes and pathways, as well as associations with self-identified race and ethnicity and overall survival in US veterans.

**Design, Setting, and Participants:**

This retrospective cohort study compared alteration frequencies between non-Hispanic Black and White men who underwent NGS testing from January 23, 2019, to November 2, 2023, adjusted by NGS analyte and clinicopathologic covariates. The analytic data file was locked on December 8, 2023. NGS testing was performed through the Department of Veterans Affairs (VA) National Precision Oncology Program, part of the largest near-equal access integrated health care system in the US.

**Exposures:**

Pathogenic alterations identified by NGS testing with a commercially available NGS platform.

**Main Outcomes and Measures:**

The primary outcome consisted of alteration frequencies in individual genes, actionable targets, and canonical prostate cancer pathways. Associations between alteration frequency and race and ethnicity as well as survival were also examined.

**Results:**

A total of 5015 veterans with mPCa who underwent NGS were included (1784 non-Hispanic Black [35.6%] and 3231 non-Hispanic White [64.4%]; mean [SD] age, 67.4 [9.0] years). Non-Hispanic Black veterans were younger, had higher prostate-specific antigen levels at diagnosis, were less likely to report Agent Orange exposure, and resided in more deprived neighborhoods compared with non-Hispanic White veterans. Nine of the top 10 most commonly altered genes were the same in non-Hispanic Black and non-Hispanic White veterans; however, the frequencies of alterations varied by race and ethnicity. Non-Hispanic Black race and ethnicity was associated with higher odds of genomic alterations in *SPOP* (odds ratio [OR], 1.7; 95% CI, 1.2-2.6) as well as immunotherapy targets (OR, 1.7; 95% CI, 1.1-2.5) including high microsatellite instability status (OR, 3.1; 95% CI, 1.1-9.4). Furthermore, non-Hispanic Black race and ethnicity was associated with lower odds of genomic alterations in the *AKT/PI3K* pathway (OR, 0.6; 95% CI, 0.4-0.7), androgen receptor axis (OR, 0.7; 95% CI, 0.5-0.9), and tumor suppressor genes (OR, 0.7; 95% CI, 0.5-0.8). Cox proportional hazards modeling stratified by race and ethnicity found that alterations in tumor suppressor genes, including *TP53,* were associated with shorter overall survival in both non-Hispanic Black (hazards ratio [HR], 1.54; 95% CI, 1.13-2.11) and non-Hispanic White (HR, 1.52; 95% CI, 1.25-1.85) veterans.

**Conclusions and Relevance:**

This retrospective clinical genomic profiling cohort study with a large total and proportional representation of non-Hispanic Black men with mPCa reported significant differences in alteration frequencies from key oncogenic pathways but similar survival rates in the near equal-access VA health care setting. This analysis suggests the utility of genomic testing for identifying candidates irrespective of race and ethnicity for precision oncology treatments, which could contribute to equitable outcomes in patients with mPCa.

## Key Points

**Question:**

How do actionable and nonactionable alteration frequencies compare between non-Hispanic Black and non-Hispanic White US veterans with metastatic prostate cancer, and what association do these alterations have with survival?

**Findings:**

In this cohort study of 5015 participants, non-Hispanic Black race and ethnicity was associated with higher odds of genomic alterations in *SPOP* and immunotherapy targets, including high microsatellite instability status, whereas non-Hispanic White race and ethnicity was associated with higher odds of genomic alterations in the *AKT/PI3K* pathway, androgen receptor axis, and tumor suppressor genes. *TP53* alterations were similarly associated with poor survival in both non-Hispanic Black and White men.

**Meaning:**

These findings reinforce the utility of genomic testing for identifying candidates, irrespective of race and ethnicity, for precision oncology treatments.

## Teaser

This cohort study evaluates the frequency of commonly reported genomic alterations as well as associations with overall survival after adjusting for patient demographic, clinical, pathological, and social determinants of health indices among non-Hispanic Black and White veterans with metastatic prostate cancer.

## Introduction

The goal of precision oncology is to personalize prognostication and treatment for individual patients and to identify candidates for life-prolonging targeted therapies. Access to next-generation sequencing (NGS) is essential to identify candidates for precision oncology approaches who are diagnosed with metastatic prostate cancer (mPCa), and national guidelines strongly recommend NGS in this heterogeneous patient population.^1,2^

Racial disparities in PCa incidence and outcomes are well documented,^3,4,5,6^ with non-Hispanic Black patients exhibiting an approximately 2.5-fold increase in likelihood of death from PCa compared with non-Hispanic White patients. While unequal access to evidence-based treatments is an important factor in this disparity, additional factors are also at play. For example, in the near-universal access Veterans Affairs (VA) health care system, self-identified non-Hispanic Black veterans still experience a higher incidence of localized and metastatic PCa compared with non-Hispanic White veterans,^7,8^ Survival differences in this unique population of non-Hispanic Black men remain unclear, especially in those with advanced disease.^7,9^ Non-Hispanic Black men are also notably underrepresented in precision medicine cohorts,^10^ such as those reporting genomic alteration frequencies in metastatic disease,^11,12^ in part due to lower rates of NGS testing in these populations.^13^

The National Precision Oncology Program (NPOP) within the Veterans Health Administration provides NGS for veterans with metastatic cancers^14^ and represents an unparalleled platform for assessing the landscape of alteration rates in mPCa across self-identified racial and ethnic groups in the diverse US veteran population. In a previous unadjusted analysis,^15^ differential rates of actionable alterations based on self-identified race and ethnicity could not be identified. However, there remains an unmet need to understand whether genomically defined actionable therapeutic targets differ by self-identified race and how these differences could influence treatment decisions and outcomes. Herein, we report an analysis of alteration rates in both individual genes as well as hallmark PCa pathways and actionable gene groupings in non-Hispanic Black and non-Hispanic White veterans diagnosed with mPCa within the VA health care system. Our goal was to evaluate the frequency of commonly reported genomic alterations as well as associations with overall survival after adjusting for NGS analyte as well as patient demographic, clinical, pathological, and social determinants of health indices.

## Methods

### Patients

This retrospective cohort study included US veterans who underwent somatic tumor testing after diagnosis with mPCa between January 23, 2019, and November 2, 2023. The analytic data file was locked on December 8, 2023. Patient self-identified race and cancer outcomes were compared among patients with a given pathogenic alteration (cases) vs those without a given alteration (controls). DNA sequencing data from tissue or plasma were eligible for inclusion. Tissue biospecimens included prostate biopsy specimens, radical prostatectomy specimens, and prostate cancer metastases. All specimens were sequenced with commercially available platforms (FoundationOne CDx or FoundationOne Liquid CDx; Foundation Medicine). When multiple specimens from the same patient were sequenced (n = 232), the first sequenced specimen was selected for analysis.^16^ Genomic and clinicopathologic data elements from the VA Corporate Data Warehouse^17^ were coanalyzed under the VA Central Institutional Review Board–approved RESOLVE (Rate Elements Skewing Outcomes Linked to Veteran Equity) substudy of the VA-MAPP (Multi-OMICs Analysis Platform for Prostate Cancer) data repository study, from which nongenomic data elements were imported. Details on the linkage of NPOP data with clinical data elements have been reported previously.^18,19^ This study adhered to Strengthening the Reporting of Observational Studies in Epidemiology (STROBE) reporting guideline for observational cohort studies. Owing to the scale, retrospective design, and deidentified nature of the nongenomic data elements, informed consent was waived.

Race and ethnicity were self-reported by veterans, and categories for the purpose of this analysis included non-Hispanic Black or African American and non-Hispanic White. For 1 patient, race was known but the sample sequenced was not, so this patient was excluded from analyses involving tissue type.

### Genomic Analyses

Short variant, copy number alteration, and rearrangement variant calls were provided by the NGS platform to NPOP, annotated, and classified by oncogenicity (eTable 1 in Supplement 1).^16^ Oncogenic alteration rates were determined for each gene and for groupings of genes into hallmark oncogenic pathways in prostate cancer (eTable 2 in Supplement 1). These included mismatch repair (MMR) deficiency genes,^20^ immunotherapy targets, DNA repair pathways, prostate cancer-specific Poly (adenosine diphosphate–ribose) polymerase inhibitor (PARPi) targets, the *AKT/PI3K* pathway, androgen receptor (AR)–signaling pathways, tumor suppressor pathways, and other known targetable pathways. Alterations affecting fewer than 11 patients were not reported to protect veteran privacy.

### Statistical Analysis

Alteration frequencies were calculated by dividing the number of alterations identified for a specific gene or gene grouping by the total number of patients tested for that gene or gene grouping. Fisher exact testing was used to compare alteration frequencies based on veteran self-identified race and ethnicity and specimen tested in our univariate analysis. To account for multiple comparisons, *P* values underwent multiple testing correction for both the individual gene and gene grouping analysis, using the Benjamini and Hochberg method.^21^ The confidence level for the 95% CI was .05.

For genes where significant differences in alteration frequencies were identified between non-Hispanic Black and non-Hispanic White veterans, multivariable logistic regression was then performed on those individual genes and their associated pathways to identify differential associations between race and alteration frequencies among all samples tested. Covariates that were adjusted for included sample type, age at diagnosis, age at sample collection, de novo metastatic status, castration resistance status, prostate-specific antigen (PSA) level at diagnosis, Gleason grade, military exposures, tumor histology, Charlson Comorbidity Index, Area Deprivation Index (ADI; calculated as previously described^22^), smoking status, and marital status. To evaluate the association of genomic alterations with survival, overall survival estimates were evaluated using a Cox proportional hazards regression model, stratified by race and adjusted for the same previously mentioned covariates.

## Results

Among a total of 6498 patients with an mPCa diagnosis who received NGS testing through NPOP, 5559 patients self-identified as either non-Hispanic Black or non-Hispanic White. After excluding 544 patients with nonmetastatic disease using a natural language processing algorithm,^23^ and 1 patient for lack of annotation of tissue type (primary vs metastatic site), sequencing data from 5015 veterans with mPCa were available for analysis (eFigure 1 in Supplement 1).

Patient and disease characteristics from the time of diagnosis are shown in the Table. Patient and disease characteristics in relation to the time of NGS specimen collection are shown in eTable 3 in Supplement 1. Mean (SD) age of the entire cohort at diagnosis was 67.4 (9.0) years. A total of 1784 patients (35.6%) self-identified as non-Hispanic Black and 3231 (64.4%) as non-Hispanic White. Non-Hispanic Black veterans were significantly younger at the time of diagnoses (mean [SD] age, 64.8 [8.8] vs 68.8 [8.8] years; *P* < .001), presented with higher PSA levels at diagnosis (997 [55.9%] vs 1592 [49.3%] with a PSA >20 ng/mL; *P* < .001), were less likely to have Agent Orange exposure (293 [16.4%] vs 893 [27.6%]; *P* < .001), and were more likely to reside in state block groups with higher ADI (mean [SD] ADI, 6.42 [2.72] vs 5.23 [2.72] ; *P* < .001).

**Table.** Patient and Disease Characteristics of Self-Identified Non-Hispanic Black and Non-Hispanic White Patients With Metastatic PCa and Next-Generation Sequencing Tumor Testing

Patient and disease characteristics at diagnosis	Entire cohort (N = 5015)	Non-Hispanic Black patients (n = 1784)	Non-Hispanic White patients (n = 3231)	*P* value^a^
Age at PCa diagnosis, y				
Mean (SD)	67.4 (9.0)	64.8 (8.8)	68.8 (8.8)	<.001
Median (IQR) [range]	67 (61-73) [31-97]	64 (59-70) [31-97]	69 (63-74) [42-95]	
Age group at PCa diagnosis, No. (%), y				
18-49	93 (1.9)	59 (3.3)	34 (1.1)	<.001
50-64	1878 (37.4)	856 (48.0)	1022 (31.6)	
65-79	2553 (50.9)	764 (42.8)	1789 (55.4)	
≥80	491 (9.8)	105 (5.9)	386 (11.9)	
Year of PCa diagnosis, No. (%)				
2000-2012	1317 (26.3)	489 (27.4)	828 (25.6)	.60
2013-2018	1218 (24.3)	432 (24.2)	786 (24.3)	
2019-2023	2193 (43.7)	770 (43.2)	1423 (44.0)	
Pre-2000	90 (1.8)	30 (1.7)	60 (1.9)	
Unknown	197 (3.9)	63 (3.5)	134 (4.1)	
PSA value at PCa diagnosis, No. (%), ng/mL				
<1	119 (2.4)	27 (1.5)	92 (2.8)	<.001
1-4	462 (9.2)	120 (6.7)	342 (10.6)	
4-10	1188 (23.7)	401 (22.5)	787 (24.4)	
10-20	657 (13.1)	239 (13.4)	418 (12.9)	
>20	2589 (51.6)	997 (55.9)	1592 (49.3)	
Grade group closest to PCa diagnosis, No. (%)				
Grade 1	436 (8.7)	165 (9.2)	271 (8.4)	.07
Grade 2	560 (11.2)	230 (12.9)	330 (10.2)	
Grade 3	520 (10.4)	178 (10.0)	342 (10.6)	
Grade 4	940 (18.7)	345 (19.3)	595 (18.4)	
Grade 5	1565 (31.2)	543 (30.4)	1022 (31.6)	
Unknown	994 (19.8)	323 (18.1)	671 (20.8)	
Military exposure, No. (%)				
Agent Orange	1186 (23.6)	293 (16.4)	893 (27.6)	<.001
Agent Orange and Camp Lejeune	19 (0.4)	5 (0.3)	14 (0.4)	
Camp Lejeune	42 (0.8)	14 (0.8)	28 (0.9)	
None or unknown	3768 (75.1)	1472 (82.5)	2296 (71.1)	
Period of service, No. (%)				
Korean War or after	484 (9.7)	115 (6.4)	369 (11.4)	<.001
Vietnam War or after	4118 (82.1)	1447 (81.1)	2671 (82.7)	
Middle East wars	374 (7.5)	211 (11.8)	163 (5.0)	
Other	39 (0.8)	11 (0.6)	28 (0.9)	
State block group ADI at PCa diagnosis^b^				
Mean (SD)	5.66 (2.78)	6.42 (2.72)	5.23 (2.72)	<.001
Median (IQR) [range]	6.00 (3.00-8.00) [1.00-10.00]	7.00 (4.00-9.00) [1.00-10.00]	5.00 (3.00-7.25) [1.00-10.00]	
CCI at baseline				
Mean (SD)	2.3 (1.67)	2.37 (1.82)	2.27 (2)	>.99
Median (IQR) [range]	2.00 (1.00-3.00) [1.00-11.00]	2.00 (1.00-3.00) [1.00-11.00]	2.00 (1.00-3.00) [1.00-11.00]	
De novo metastasis (regional or distant)	1920 (38.3)	667 (37.4)	1253 (38.8)	.30

Abbreviations: ADI, Area Deprivation Index; CCI, Charlson Comorbidity Index; PCa, prostate cancer; PSA, prostate-specific antigen.

SI converstion factor: To convert PSA to micrograms per liter, multiply by 1.

^a^ Calculated using χ^2^ test.

^b^ Calculated as previously described in Wadhwa et al.^22^

In an unadjusted analysis of all NGS analyte results combined, non-Hispanic White veterans were significantly more likely to harbor alterations in *AKT/PI3K* pathway genes (965 [29.9%] vs 355 [19.9%]; *P* < .001), AR signaling axis genes (1429 [44.2%] vs 616 [34.5%]; *P* < .001), DNA repair genes (709 [21.9%] vs 304 [17.0%]; *P* < .001), prostate cancer–specific PARPi targets (802 [24.8%] vs 394 [22.1%]; *P* = .03), and tumor suppressor genes (1655 [51.5%] vs 682 [38.2%]; *P* < .001) (eFigure 2A and eTable 4 in Supplement 1). By contrast, non-Hispanic Black veterans were significantly more likely to harbor alterations in immunotherapy targets (195 [10.9%] vs 232 [7.2%]; *P* < .001) and MMR deficiency genes (82 [4.6%] vs 96 [3.0%]; *P* < .01) and more frequently had high microsatellite instability (MSI) (41 of 1032 [4.0%] vs 53 of 1991 [2.7%]; *P* = .03). There were no significant differences in tumor mutational burden (TMB) status or alterations in other targeted therapy pathways.

On interrogation of alteration frequency by NGS analyte (plasma vs tissue) and tissue of origin (primary prostate vs metastatic site) without stratifying for self-identified race, we observed significant differences in alterations within the hallmark pathways, with metastases significantly more likely to harbor alterations in all pathways compared with primary tumor tissue (eg, 184 of 1011 [18.2%] vs 344 of 2359 [14.6%] in DNA repair gene pathways; *P* = .01) (eTable 5 in Supplement 1). Plasma specimens were also more likely to have DNA repair alterations (485 of 1644 [29.5%] vs 344 of 2359 [14.6%]; *P* < .001), prostate cancer–specific PARPi target alterations (514 of 1644 [31.3%] vs 447 of 2359 [18.9%]; *P* < .001), and high MSI high status (21 of 102 [20.6%] vs 42 of 1988 [2.1%]; *P* < .001) but significantly less likely to harbor alterations targetable by immunotherapy (98 of 1644 [6.0%] vs 212 of 2539 [8.3%]; *P* < .001), *AKT/PI3K* pathway alterations (249 of 1644 [15.1%] vs 687 of 2539 [27.1%]; *P* < .001), and AR signaling alterations (529 of 1644 [32.2%] vs 1002 of 2539 [39.5%]; *P* < .001) compared with primary tumors. Alterations in MMR deficiency genes, tumor suppressor genes, and other targetable pathways did not differ between plasma and primary tissue. TMB status was low and similar across analytes (eFigure 2B and eTable 5 in Supplement 1).

**Figure 1.**
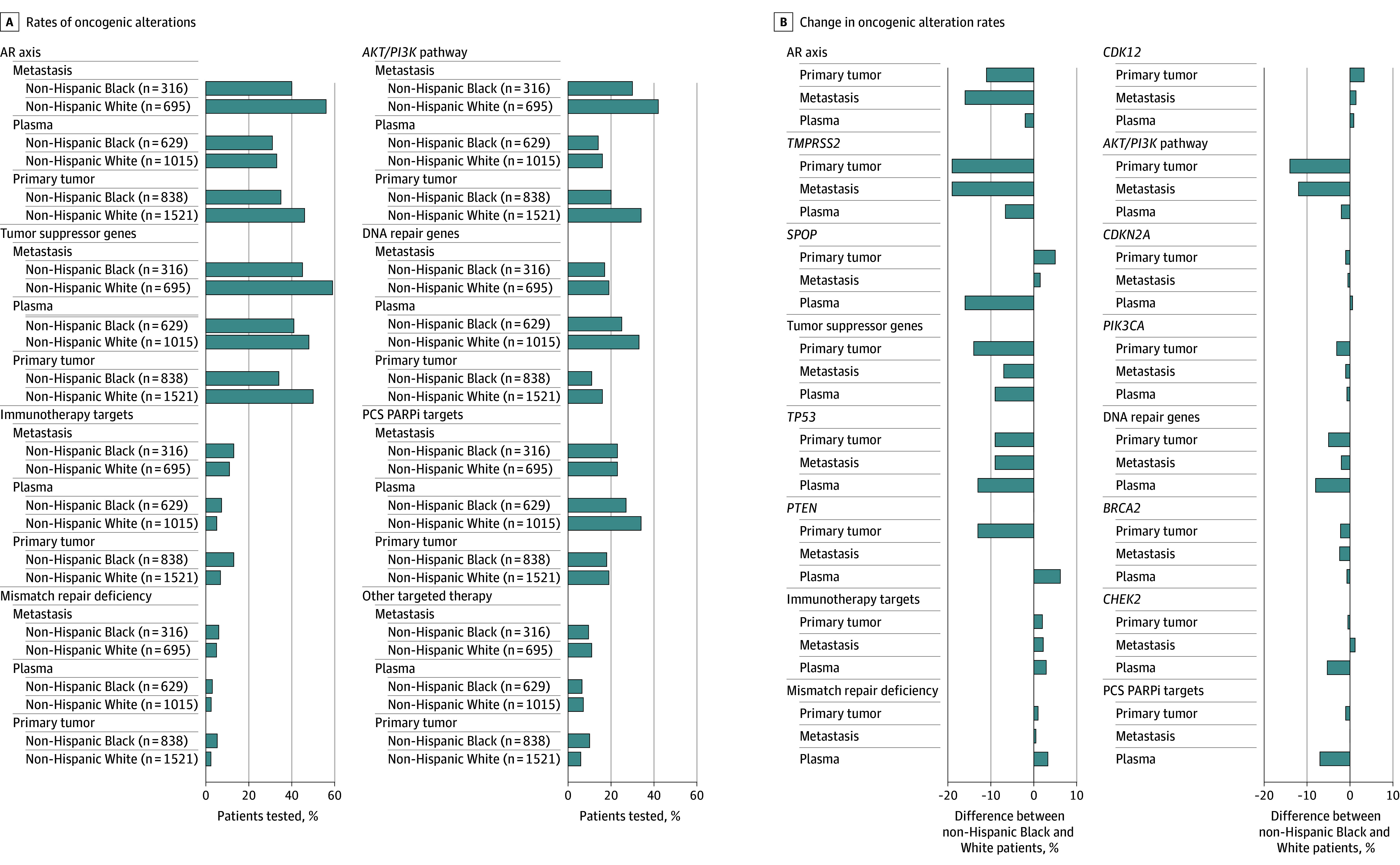
Association of Genomics, Race, and Survival in Patients With Metastatic Prostate Cancer A, Rates of oncogenic alterations in genes from cancer-related pathways are shown in 3 different tissues (primary tumor, metastatic, and plasma samples), separated by patient self-identified race. Differences in rates of oncogenic alterations between non-Hispanic Black and non-Hispanic White patients were compared by Fisher exact test for each of the 3 tissue types. B, Percentage change in oncogenic alteration rates in constituent genes within pathways are compared in non-Hispanic Black and non-Hispanic White patients in the 3 tissue types. Positive percentages indicate changes were higher in non-Hispanic Black patients; negative percentages, lower. AR indicates androgen receptor; PCS PARPi, prostate cancer–specific Poly (adenosine diphosphate–ribose) polymerase inhibitor.

Given the importance of both race and NGS analyte in alteration frequencies, we performed an aggregate comparison of alteration frequency stratified by both race and tissue type sampled, focusing on genomic alterations that were significantly different in the separate race- and analyte-based analyses described. In this analysis, 9 of the top 10 most commonly altered genes were the same in non-Hispanic Black and non-Hispanic White veterans; however, the frequencies of alterations varied by race and ethnicity. The most commonly altered genes were similar between non-Hispanic Black and non-Hispanic White veterans stratified by NGS analyte type, but the oncogenic alteration rates were significantly different between non-Hispanic Black and non-Hispanic White veterans for multiple genes (eFigures 3-5 and eTable 6 in Supplement 1). AR axis alterations were significantly more likely to be identified in non-Hispanic White samples derived from primary (707 of 1521 [46.5%] vs 295 of 838 [35.2%]; *P* < .001) and metastatic (388 of 695 [55.8%] vs 126 of 316 [39.9%]; *P* < .001) tumor tissue; however, no differences were uncovered in plasma samples (334 of 1015 [32.9%] vs 195 of 629 [31.0%]; *P* = .61). The same patterns of significance emerged for alterations in the *AKT/PI3K* pathway (Figure 1A and eTable 6 in Supplement 1). By contrast, only in sequenced plasma samples was a significant increase in prostate cancer–specific PARPi target alterations (343 of 1015 [33.8%] vs 171 of 629 [27.2%]; *P* = .01) observed in non-Hispanic White men. Alterations in DNA repair genes were more common in non-Hispanic White veterans compared with non-Hispanic Black veterans when primary tissue (249 of 1521 [16.4%] vs 95 of 839 [11.3%]; *P* = .001) and plasma (330 of 1015 [32.5%] vs 155 of 629 [24.6%]; *P* = .006) samples were tested. Regarding alterations in tumor suppressor genes, non-Hispanic White patients were significantly more likely to harbor alterations in this group of genes compared with non-Hispanic Black patients in all tissue types tested (primary tumor, 764 of 1521 [50.2%] vs 284 of 838 [33.9%]; *P* < .001; plasma, 490 of 1015 [48.3%] vs 255 of 629 [40.5%]; *P* = .009). Alterations in immunotherapy pathways (109 of 838 [13.0%] vs 103 of 1521 [6.8%]; *P* < .001), MMR deficiency (44 of 838 [5.3%] vs 36 of 1521 [2.4%]; *P* < .001), and other known targetable pathways (84 of 838 [10.0%] vs 89 of 1521 [5.9%]; *P* < .001) were all significantly more likely in non-Hispanic Black patients, but only when the primary tumor was tested (Figure 1A and eTable 6 in Supplement 1).

When considering alterations in the individual genetic components of the previously described oncogenic pathways that were significantly different by race, it becomes clear that AR signaling differences were likely associated with alteration rates in *TMPRSS2* rather than *SPOP* (Figure 1B). *TP53* and *PTEN* alterations appeared to contribute similarly to the lower tumor suppressor alteration frequency in non-Hispanic Black veterans, while MMR deficiency and *CDK12* alterations appeared to contribute similarly to the increase in immunotherapy targets in non-Hispanic Black individuals (Figure 1B).

**Figure 2.**
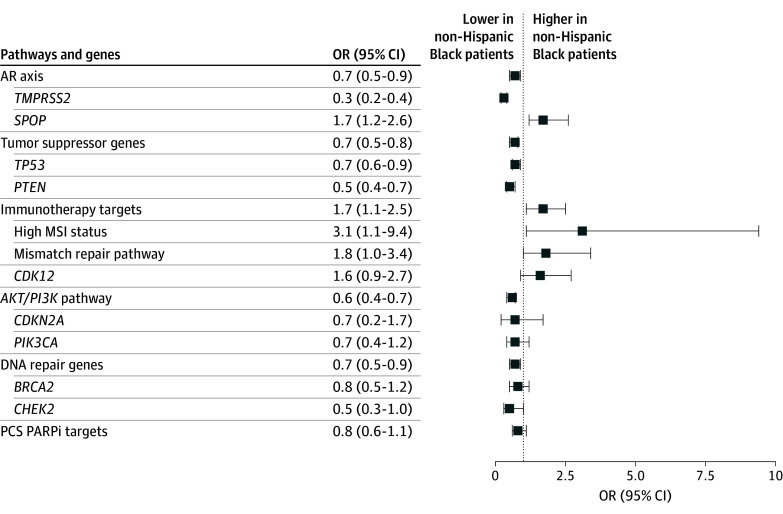
Association of Race and Alteration Frequency in Patients With Metastatic Prostate Cancer Multivariable logistic regression analysis was used to evaluate the association of oncogenic alterations in pathways and genes in those pathways with self-identified race, controlling for multiple clinical factors. Pathways and genes were tested if a statistically significant difference in rates was found between non-Hispanic Black and non-Hispanic White patients in univariate analyses. Numeric values for the odds ratios (ORs) and 95% CIs are found in eTable 7 in Supplement 1. AR indicates androgen receptor; MSI, microsatellite instability; OR, odds ratio; and PCS PARPi, prostate cancer–specific Poly (adenosine diphosphate–ribose) polymerase inhibitor.

Since analyte and race can both impact alteration frequencies, multivariable analysis was pursued to better understand the association between race and alteration frequency. After multivariable adjustment for patient and tumor-related characteristics, including NGS analyte, clinicopathologic features, and social determinants of health (SDOH) covariates, alteration frequencies in non-Hispanic Black veterans were found to be significantly lower in AR axis genes (odds ratio [OR], 0.7; 95% CI, 0.5-0.9; *P* < .001), tumor suppressor genes (OR, 0.7; 95% CI, 0.5-0.8; *P* < .001), DNA repair genes (OR, 0.7; 95% CI, 0.5-0.9; *P* < .001), and *AKT/PI3K* pathway genes (OR, 0.6; 95% CI, 0.4-0.7; *P* < .001), but significantly elevated in *SPOP* (OR, 1.7; 95% CI, 1.2-2.6; *P* = .006) and targets for immunotherapy (OR, 1.7; 95% CI, 1.1-2.5; *P* = .02), including high MSI status (OR, 3.1; 95% CI, 1.1-9.4; *P* = .03) (Figure 2 and eTable 7 in Supplement 1).

**Figure 3.**
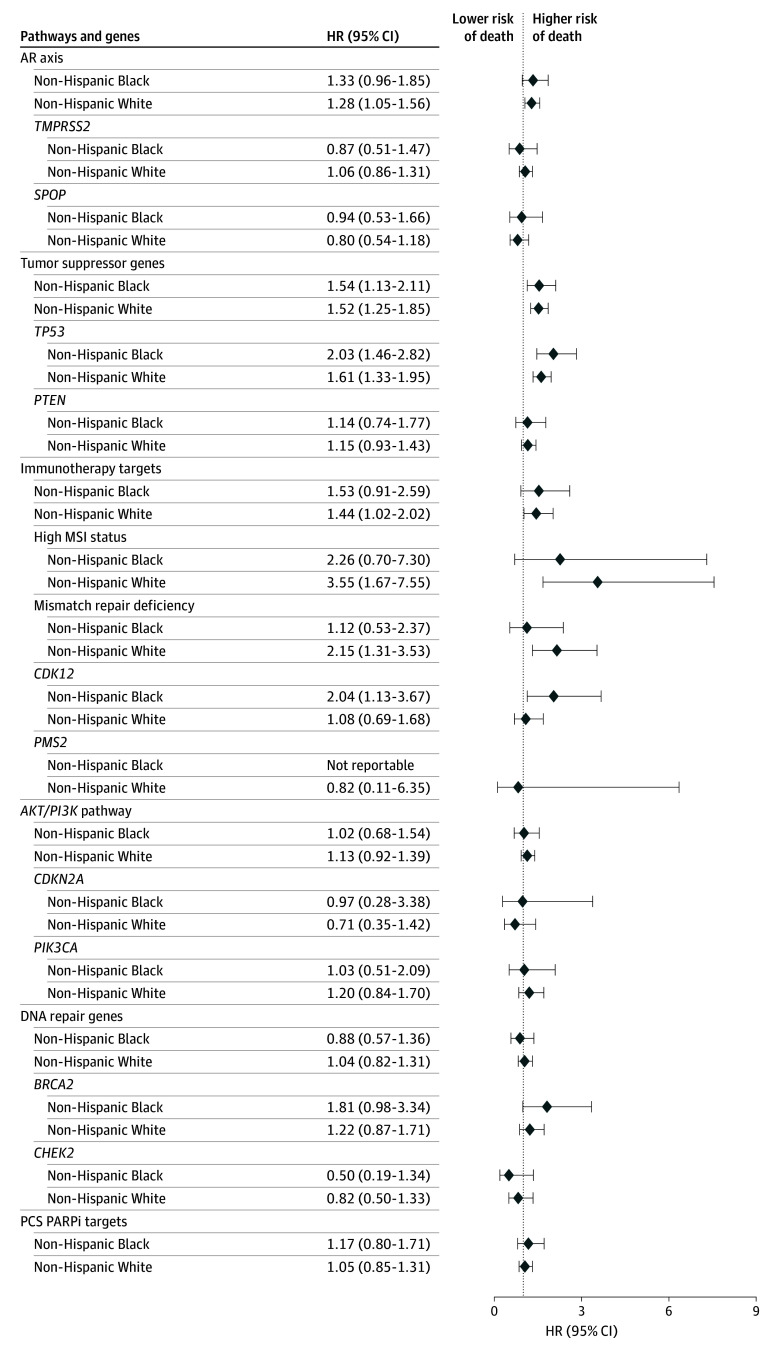
Association of Genomics, Race, and Survival in Patients With Metastatic Prostate Cancer Cox proportional hazards modeling was used to test the association of oncogenic alterations in pathways and genes in those pathways with overall survival, controlling for clinical factors and stratified by race. Numeric values for the hazard ratios (HRs) and 95% CIs are found in eTable 8 in Supplement 1. AR indicates androgen receptor; MSI, microsatellite instability; and PCS PARPi, prostate cancer–specific Poly (adenosine diphosphate–ribose) polymerase inhibitor.

Survival has been previously reported to be similar in non-Hispanic Black veterans compared with non-Hispanic White veterans who receive their care within the Veterans Health Administration.^7,24^ In our adjusted Cox proportional hazards model for overall survival, tumor suppressor alterations driven by *TP53* alterations (hazard ratio [HR], 1.52; 95% CI, 1.25-1.85), immunotherapy targets driven by MMR deficiency and MSI high status (HR, 144; 95% CI, 1.02-2.02), and AR axis alterations (HR, 1.28; 95% CI, 1.05-1.56) increased the hazard of death in non-Hispanic White veterans, whereas tumor suppressor alterations driven by *TP53* alterations (HR, 1.54; 95% CI, 1.13-2.11) and *CDK12* alterations (HR, 2.04; 95% CI, 1.13-3.67) both increased the hazard of death in non-Hispanic Black veterans (Figure 3 and eTable 8 in Supplement 1).

## Discussion

This cohort study describes alteration frequencies in key PCa pathways and genes, including those that are known to be targetable with precision oncology interventions in a large cohort of non-Hispanic Black and non-Hispanic White veterans with mPCa. Absolute and proportional (35.6%) representation of non-Hispanic Black individuals in this study was markedly higher than in previous reports.^11,12,25,26^ The observed increased frequency of immunotherapy targets in non-Hispanic Black individuals is of interest because of potential actionability in this population and underscores the continued need for equitable application of precision medicine efforts in mPCa, as this could be one of many factors that can mitigate disparities in PCa outcome.

Associations between responsiveness to immunotherapy and patient race have been previously reported from PROCEED registry data,^27^ where overall survival was shown to be significantly higher in Black men with castrate-resistant mPCa who were treated with the first US Food and Drug Administration–approved cellular immunotherapy, sipuleucel-T. Examples of associations between high MSI status and complete response to sipuleucel-T have also been reported in the literature,^28^ and high MSI status is also a marker for responsiveness to programmed cell death 1 axis inhibition,^29^ such that the higher frequency of alterations in immunotherapy targets among non-Hispanic Black men, driven by higher MSI status, may contribute to enhanced immunogenicity of tumors in this population. Consequently, this heightened immunogenicity could lead to more favorable responses to immunotherapy. Of note, TMB, a known factor associated with response to checkpoint inhibition, did not differ significantly between non-Hispanic White and non-Hispanic Black veterans. Our finding that non-Hispanic White men with mPCa have a higher frequency of *TP53* alterations aligns with the hypothesis proposed by Halabi and colleagues,^30^ suggesting an interaction between an intact *TP53* pathway and the efficacy of docetaxel as a plausible explanation for the improved outcomes observed in Black men treated with the wild-type p53–dependent drug, docetaxel.

In addition to our findings related to immunotherapy targets, non-Hispanic White individuals were more likely to harbor alterations in AR signaling than non-Hispanic Black individuals. This may contribute to decreased responsiveness to AR signaling inhibitors in non-Hispanic White compared with non-Hispanic Black patients, an observation that has been reported in individuals with castrate-resistant disease who received abiraterone acetate as first-line therapy.^31^ This work also raises the possibility that while canonical AR activity may be lower in non-Hispanic Black patients, the activity of noncanonical AR pathways may be increased in non-Hispanic Black individuals, a possibility that requires further research.

Our report also underscores the fact that the source of tumor DNA (ie primary vs metastatic tissues vs plasma) submitted for NGS testing can influence the frequency of specific alterations and confound comparisons between race and thus remains an important control for analyses of this nature. Accordingly, sequencing results used by clinicians should always be considered in the context of the analyte tested. For example, our findings highlight that plasma evaluating cell-free DNA may not be as sensitive as tumor tissue for the identification of actionable immunotherapy targets; indeed, less than 10% of the plasma samples tested in this study reported microsatellite status. Plasma samples may also overreport frequencies of alterations in actionable DNA damage repair (DDR) genes (eg, of *ATM* or *CHEK2*), which may be derived from clonal hematopoiesis, especially in older patients^32^ such as veterans with mPCa. Conversely, alterations in *CDKN2A*, *ERBB2*, *PTEN*, *RB1*, and *TMPRSS2* were found at lower frequencies in plasma samples, likely due to challenges identifying copy number variants and rearrangements in cell-free DNA vs tissue.

It has also been previously reported that many actionable PCa alterations, including DDR alterations, are truncal in nature^33^ and that their frequencies are therefore similar in both primary and metastatic tissue. Our work is largely consistent with this observation, and while metastases were significantly more likely to yield actionable alterations in DNA repair genes than primary tissue, this difference was small (18.2% vs 14.6%).

Prior unadjusted analyses^15,34^ have suggested that actionable alterations appear at similar rates between non-Hispanic Black and non-Hispanic White veterans, whereas the present larger adjusted analysis confirms DDR alteration frequencies are lower in non-Hispanic Black individuals, consistent with similar findings in the germline. Our adjusted analysis also suggests that immunotherapy targets are more frequent in non-Hispanic Black men. While this was certainly the case for high MSI status, the picture is less clear for *CDK12* alterations, which are known to be associated with aggressive PCa phenotypes. Previous work that includes some of the present investigators^15^ revealed similar *CDK12* alteration rates between non-Hispanic Black and non-Hispanic White veterans, and while there was a higher frequency of these alterations in non-Hispanic Black veterans in our univariate analysis, this difference disappeared after multivariable adjustment. Nevertheless, *CDK12* may remain a consequential target for non-Hispanic Black men with mPCa, and we echo others^35,36^ in highlighting the fact that therapeutic targeting of *CDK12* may serve as an avenue for improving outcomes for non-Hispanic Black patients.

Overall, this work emphasizes the notion that personalized precision medicine–driven approaches will be essential to maximally leverage our understanding of a patient’s specific tumor biology and to improve oncologic outcomes. This is desperately needed, as non-Hispanic Black men have consistently been shown to exhibit more aggressive PCa phenotypes as well as more than twice^37^ the incidence and prevalence of PCa across all disease states compared with non-Hispanic White patients. Even in scenarios where nontargetable alterations are identified (such as the case of tumor suppressor alterations including *TP53*), knowledge of the deleterious implications of these alterations on survival (consistent with previously reported work from Velez and colleagues^38^) can have wide implications for the management of mPCa among both non-Hispanic Black and non-Hispanic White men and result in changes in treatment. Specifically, given the observation that high-risk alterations (including *TP53, RB1*, and *PTEN*) can promote lineage plasticity, resulting in radiographic progression in the absence of PSA progression,^39,40^ the presence of these pathogenic alterations may have implications for the optimal cadence and modality of disease monitoring. Knowledge of these alterations may also assist in patient selection for metastasis-directed therapy with stereotactic body radiotherapy in the oligometastatic setting, given the results of a pooled analysis^41^ that found a high-risk mutational signature consisting of alterations in *BRCA1/2*, *ATM*, *TP53*, or *RB1* was independently associated with relative benefit and prognostic for response to metastasis-directed therapy. Last, this work implicitly advocates for the intentional design of precision medicine studies that do not exclude groups of patients who have been historically underrepresented in clinical trials, such as non-Hispanic Black men.

### Limitations

A limitation of our analysis is the lack of matched germline data for these patients, which complicates interpretation of plasma results. Additionally, by analyzing the first specimen sent for NGS testing, there is a possibility of false-negative results reported among an inconsequential number of patients who had multiple samples sent for NGS testing over the course of their disease, although the low frequency of these false-negative results would not change our results or their interpretation in any meaningful way. Furthermore, survivorship bias (ie, excluding patients with metastatic disease who did not live long enough to undergo NGS) is an inherent feature of our analysis, which may inadvertently exclude the most aggressive mPCa phenotypes. Last, the US veteran population (including both non-Hispanic Black and non-Hispanic White individuals) served by the VA health care system represents a benefit population with health care outcomes and biological profiles that may not generalize to the population at large.

## Conclusions

In this retrospective cohort study of 5015 US veterans with mPCa who underwent NGS, we found that alteration frequencies in several hallmark oncogenic pathways varied by race and that the association between specific alterations (eg, *CDK12*, AR axis, MMR deficiency) and survival, when stratified by race, was also variable. Importantly, we did not identify any genomic alterations or biomarkers that should not be tested in PCa based on patient self-identified race. Ultimately, this work emphasizes that precision oncology enables the individualization of treatment decisions without having to rely on imprecise characteristics such as self-identified race. While individuals may exhibit different biological aggressiveness and associated outcomes from PCa treatment, precision-based testing and treatment approaches remain critical for personalizing care, optimizing outcomes, informing the design of equitable clinical trials, and narrowing disparities in outcomes for mPCa.

## Notes

Supplement 1. **eFigure 1.** Consort Diagram Demonstrating Patients Included and Excluded for Analysis **eFigure 2.** Differences in Oncogenic Pathways for Individuals With Metastatic Prostate Cancer **eFigure 3.** Heatmaps Denoting Genomic Alterations From Primary Tissue **eFigure 4.** Heatmaps Denoting Genomic Alterations From Metastatic Tissue **eFigure 5.** Heatmaps Denoting Genomic Alterations From Liquid Biopsy **eTable 1.** Categories of Oncogenic and Likely Oncogenic Variants **eTable 2.** Genes Involved in Studied Pathways **eTable 3.** Patient and Disease Characteristics of Self-Identified Non-Hispanic Black and Non-Hispanic White Patients With Metastatic Prostate Cancer in Relation to Time of NGS Specimen Collection **eTable 4.** Oncogenic Alteration Rates in All Tests in Patients With mPCa **eTable 5.** Oncogenic Alteration Rates in Primary Prostate vs Metastasis and Liquid Biopsies in Patients With mPCa **eTable 6.** Oncogenic Alteration Rates in Non-Hispanic Black vs Non-Hispanic White Patients With mPCa Stratified by Tissue Type Tested **eTable 7.** Association of Race With Alteration Frequency in mPCa **eTable 8.** Association of Overall Survival With Oncogenic Alterations in Individual Genes and Pathways

Supplement 2.
Data Sharing Statement
