# Supplementary material for: Somatic Tumor Next-Generation Sequencing in US Veterans With Metastatic Prostate Cancer
Source: JAMA Netw Open. 2025 May 12;8(5):e259119. doi: 10.1001/jamanetworkopen.2025.9119 (PMC12070238; doi:10.1001/jamanetworkopen.2025.9119)
Supplement: Supplement 1. — eFigure 1. Consort Diagram Demonstrating Patients Included and Excluded for Analysis eFigure 2. Differences in Oncogenic Pathways for Individuals With Metastatic Prostate Cancer eFigure 3. Heatmaps Denoting Genomic Alterations From Primary Tissue eFigure 4. Heatmaps Denoting Genomic Alterations From Metastatic Tissue eFigure 5. Heatmaps Denoting Genomic Alterations From Liquid Biopsy eTable 1. Categories of Oncogenic and Likely Oncogenic Variants eTable 2. Genes Involved in Studied Pathways eTable 3. Patient and Disease Characteristics of Self-Identified Non-Hispanic Black and Non-Hispanic White Patients With Metastatic Prostate Cancer in Relation to Time of NGS Specimen Collection eTable 4. Oncogenic Alteration Rates in All Tests in Patients With mPCa eTable 5. Oncogenic Alteration Rates in Primary Prostate vs Metastasis and Liquid Biopsies in Patients With mPCa eTable 6. Oncogenic Alteration Rates in Non-Hispanic Black vs Non-Hispanic White Patients With mPCa Stratified by Tissue Type Tested eTable 7. Association of Race With Alteration Frequency in mPCa eTable 8. Association of Overall Survival With Oncogenic Alterations in Individual Genes and Pathways [file jamanetwopen-e259119-s001.pdf]

Supplementary Online Content

Valle LF, Li J, Desai H, et al. Somatic tumor next-generation sequencing in US veterans with metastatic prostate cancer. *JAMA Netw Open.* 2025;8(5):e259119. doi:10.1001/jamanetworkopen.2025.9119

eFigure 1. Consort Diagram Demonstrating Patients Included and Excluded for Analysis
eFigure 2. Differences in Oncogenic Pathways for Individuals With Metastatic Prostate Cancer
eFigure 3. Heatmaps Denoting Genomic Alterations From Primary Tissue
eFigure 4. Heatmaps Denoting Genomic Alterations From Metastatic Tissue
eFigure 5. Heatmaps Denoting Genomic Alterations From Liquid Biopsy
eTable 1. Categories of Oncogenic and Likely Oncogenic Variants
eTable 2. Genes Involved in Studied Pathways
eTable 3. Patient and Disease Characteristics of Self-Identified Non-Hispanic Black and Non-Hispanic White Patients With Metastatic Prostate Cancer in Relation to Time of NGS Specimen Collection
eTable 4. Oncogenic Alteration Rates in All Tests in Patients With mPCa
eTable 5. Oncogenic Alteration Rates in Primary Prostate vs Metastasis and Liquid Biopsies in Patients With mPCa
eTable 6. Oncogenic Alteration Rates in Non-Hispanic Black vs Non-Hispanic White Patients With mPCa Stratified by Tissue Type Tested
eTable 7. Association of Race With Alteration Frequency in mPCa
eTable 8. Association of Overall Survival With Oncogenic Alterations in Individual Genes and Pathways

This supplementary material has been provided by the authors to give readers additional information about their work.

© 2025 Valle LF et al. *JAMA Network Open*.

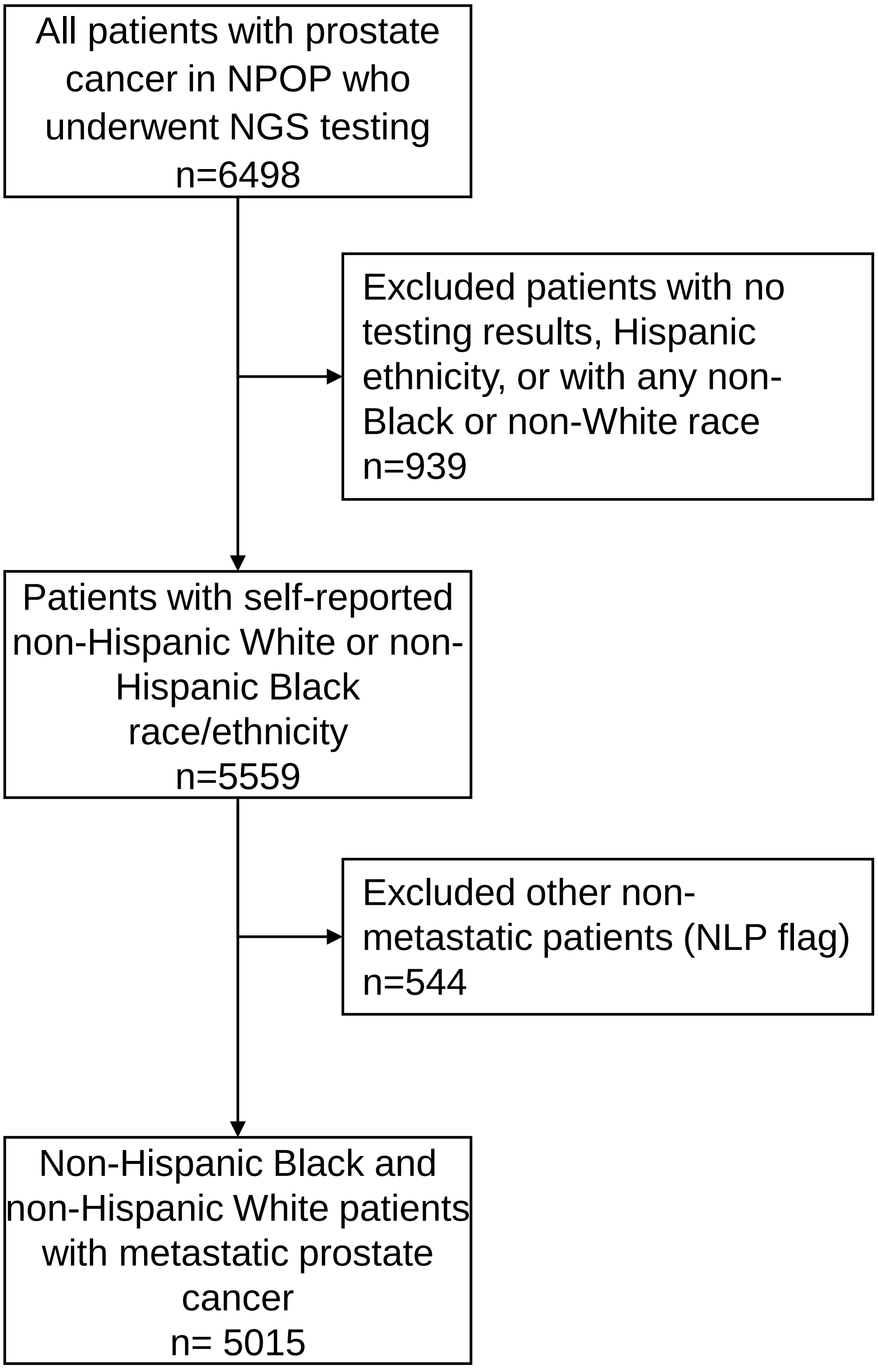

eFigure 1: Consort Diagram demonstrating patients included and excluded for analysis. NLP = natural language processing.

© 2025 Valle LF et al. *JAMA Network Open*.

a.

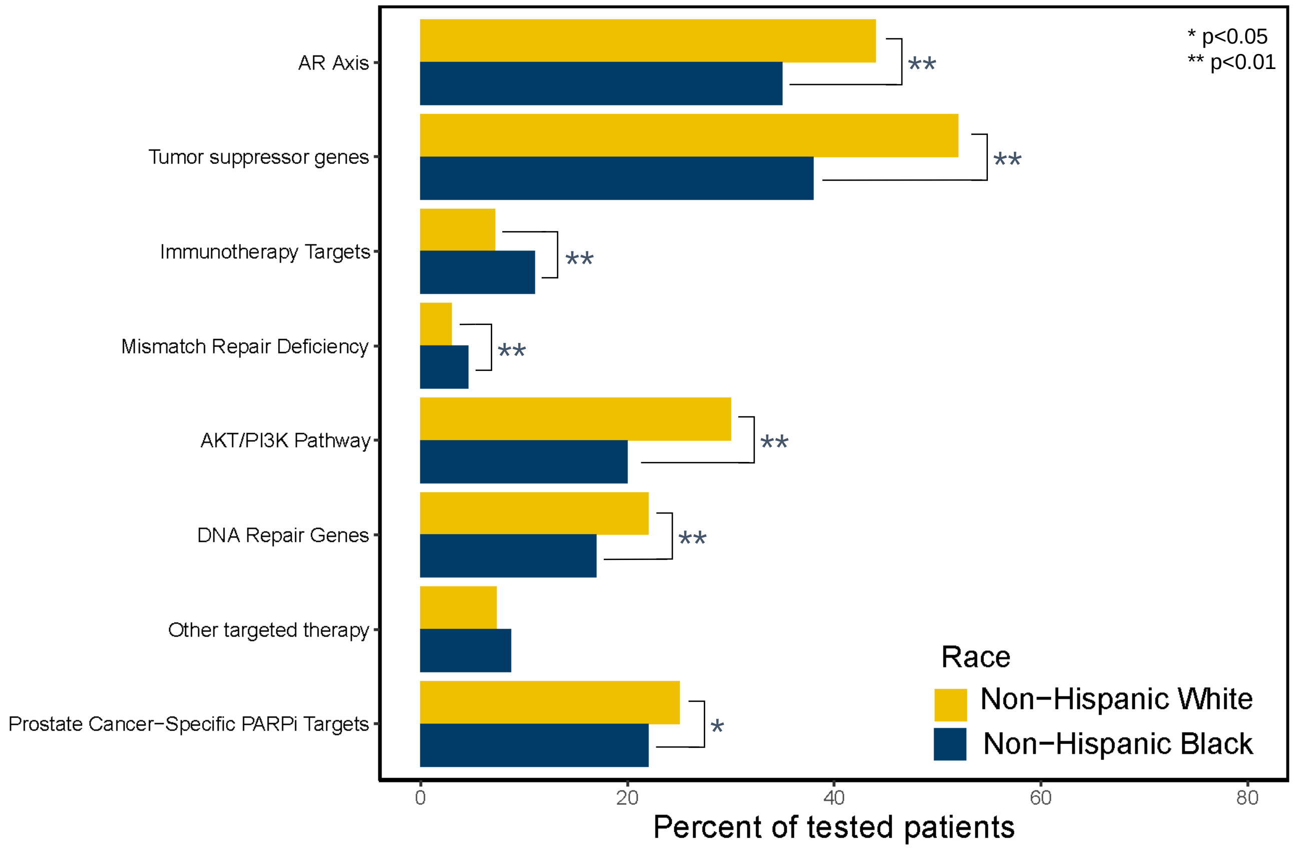

b.

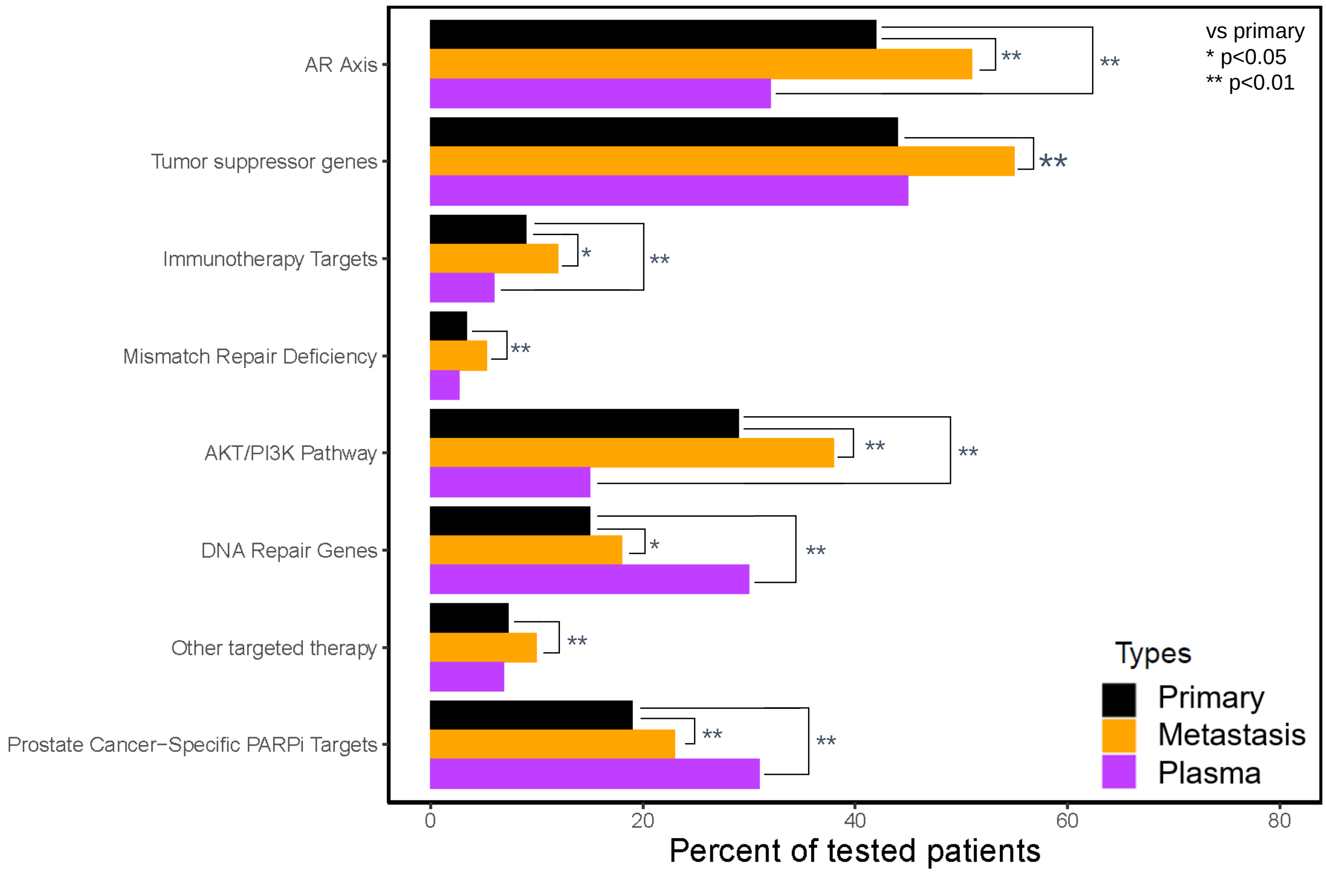

eFigure 2: Differences in oncogenic pathways for individuals with metastatic prostate cancer organized by A. patient self-identified race and by B. tissue analyzed for NGS testing. Genes involved in these pathways are listed in the Supplemental tables. NHB = non-Hispanic Black; NHW = non-Hispanic White, AR = androgen receptor, PCS = prostate cancer specific, PARPi = PARP inhibitor.

© 2025 Valle LF et al. *JAMA Network Open.*

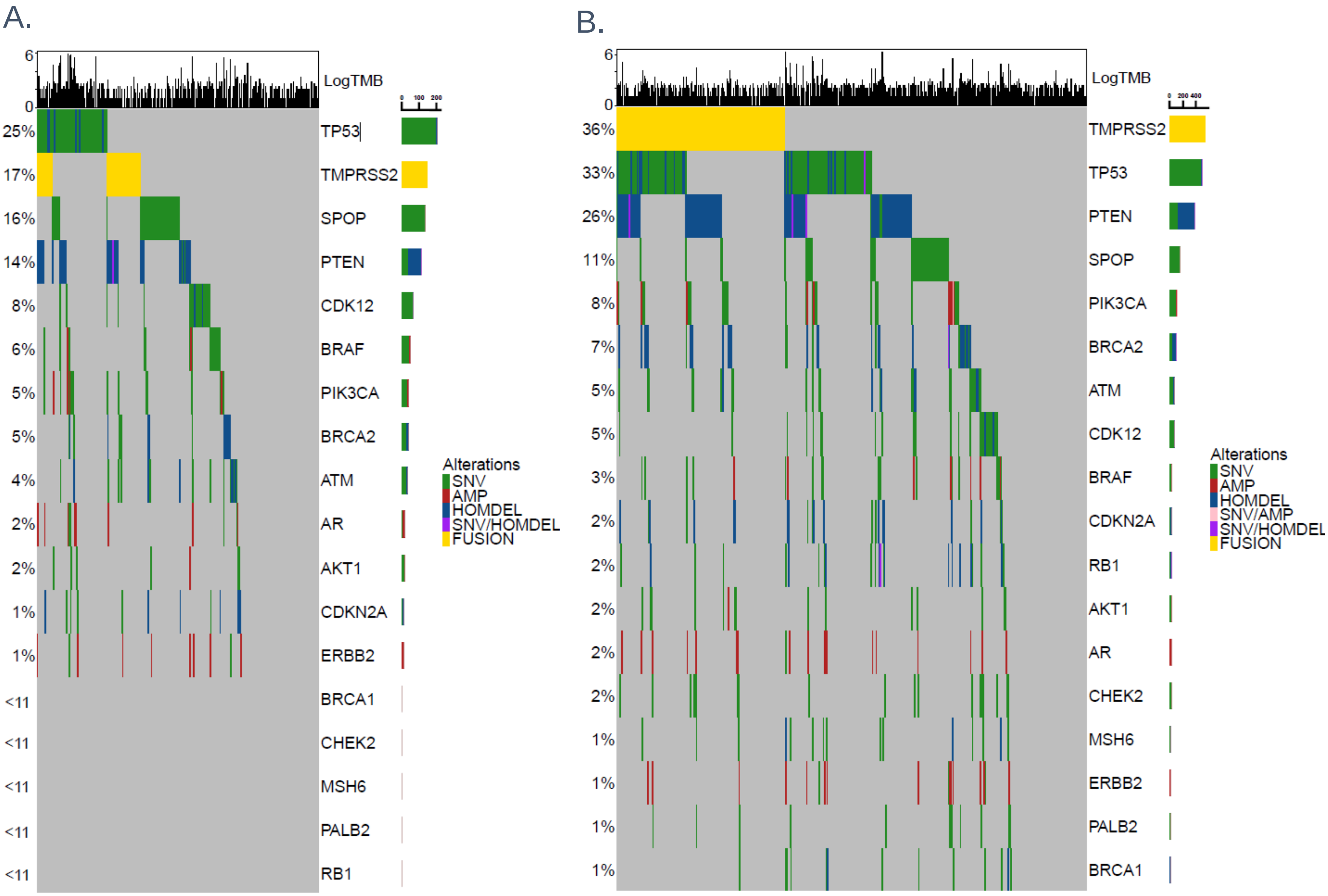

eFigure 3: Heatmaps denoting genomic alterations from primary tissue in A) NHB (n=838) and B) NHW (n=1521) Veterans.

© 2025 Valle LF et al. *JAMA Network Open*.

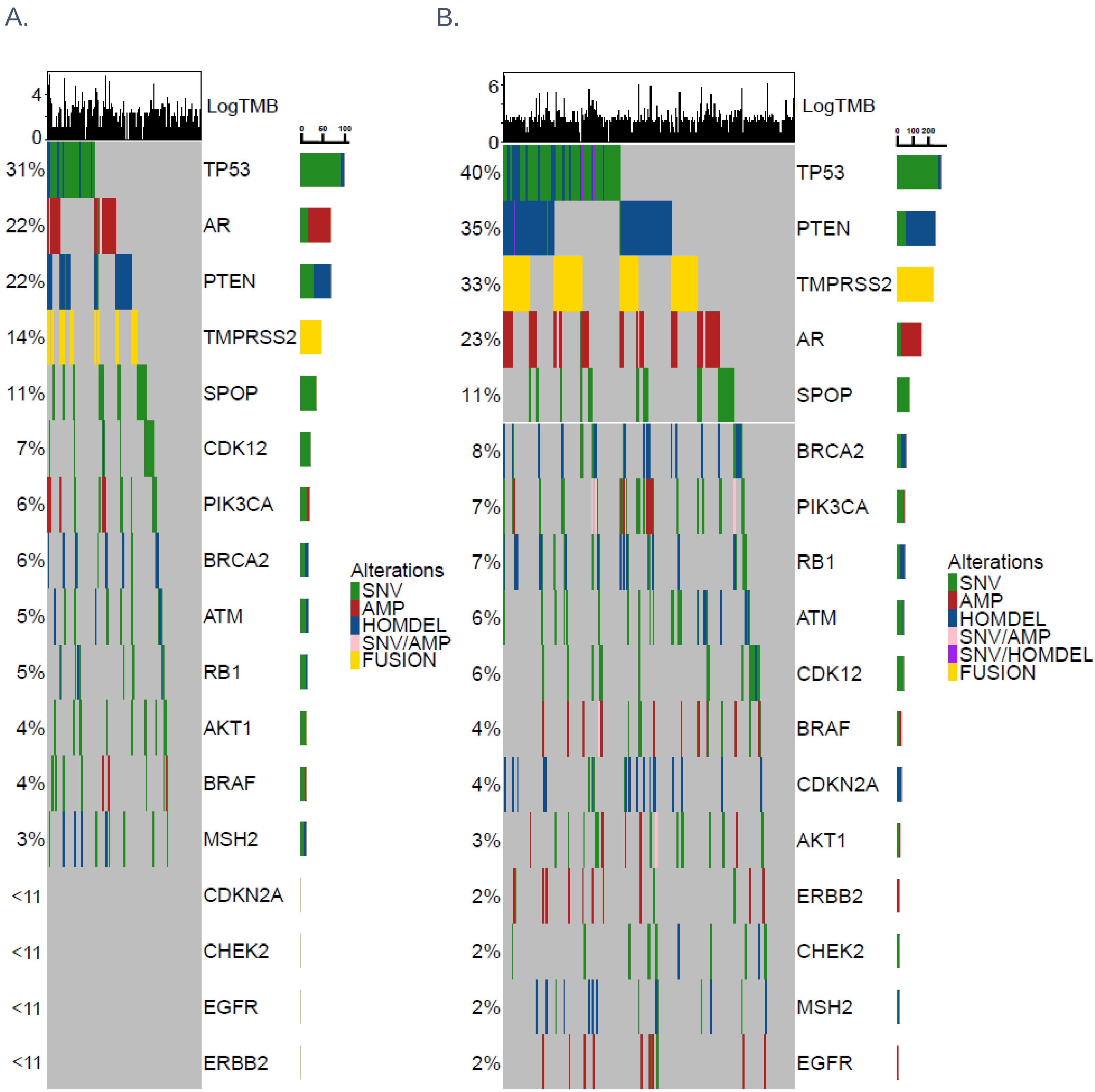

eFigure 4: Heatmaps denoting genomic alterations from metastatic tissue in A) NHB (n=316) and B) NHW (n=695) Veterans.

© 2025 Valle LF et al. *JAMA Network Open*.

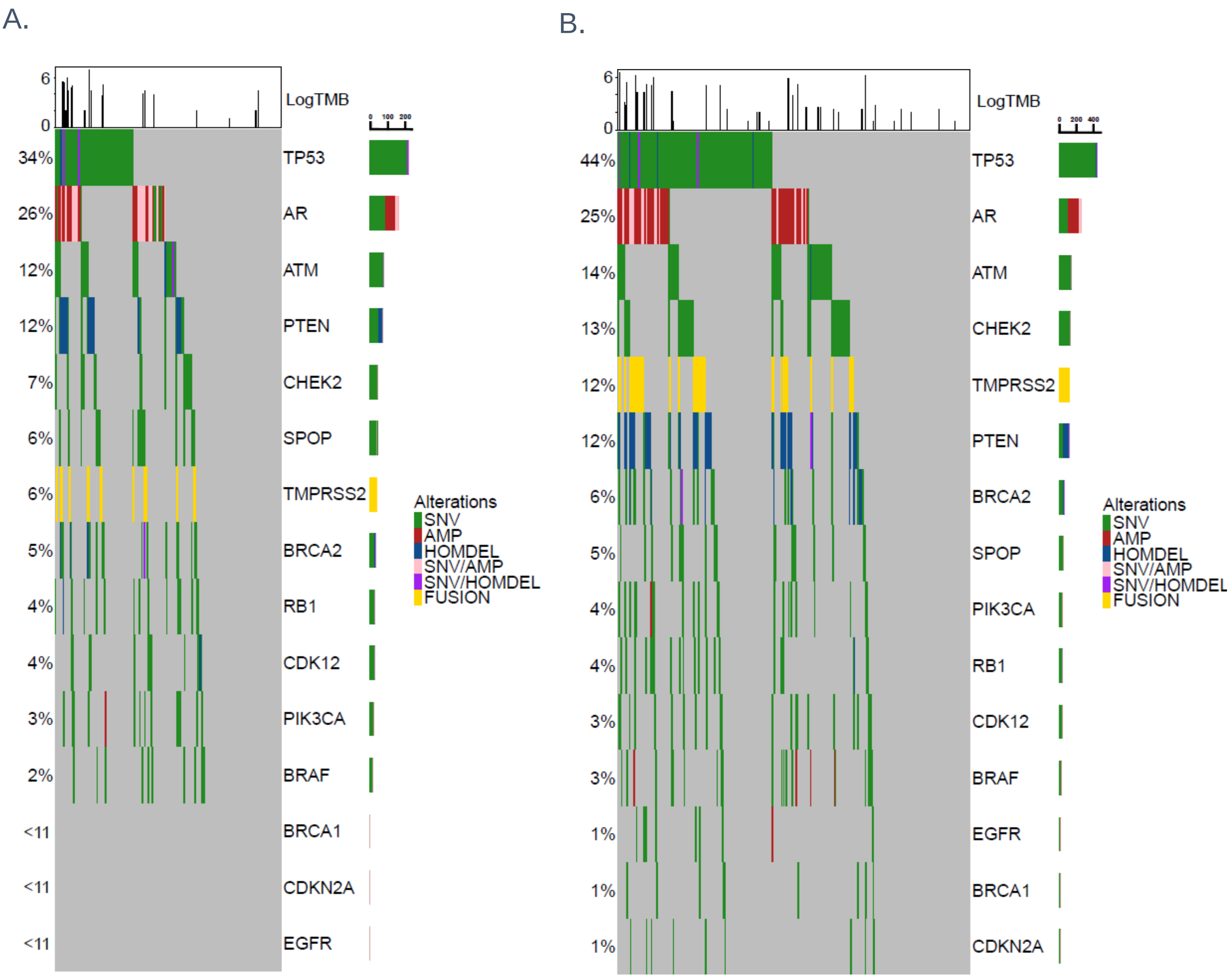

eFigure 5: Heatmaps denoting genomic alterations from liquid biopsy in A) NHB (n=629) and B) NHW (n=1015) Veterans.

© 2025 Valle LF et al. *JAMA Network Open*.

eTable 1: Categories of Oncogenic/Likely Oncogenic Variants

Gene	Gene Type	Short Variants Status	Copy Number Alterations	Rearrangement Type
AR	Oncogene	known or likely	contains "amplification"	
SPOP	Tumor Suppressor	known or likely	contains "loss" or "deletion"	deletion
TMPRSS2	Oncogene	known or likely		fusion
RB1	Tumor Suppressor	known or likely	contains "loss" or "deletion"	deletion
TP53	Tumor Suppressor	known or likely	contains "loss" or "deletion"	deletion
PTEN	Tumor Suppressor	known or likely	contains "loss" or "deletion"	deletion
AKT1	Oncogene	known or likely	contains "amplification"	
CDKN2A	Tumor Suppressor	known or likely	contains "loss" or "deletion"	deletion
PIK3CA	Oncogene	known or likely	contains "amplification"	
MLH1	Tumor Suppressor	known or likely	contains "loss" or "deletion"	deletion
MSH2	Tumor Suppressor	known or likely	contains "loss" or "deletion"	deletion
MSH6	Tumor Suppressor	known or likely	contains "loss" or "deletion"	deletion
PMS2	Tumor Suppressor	known or likely	contains "loss" or "deletion"	deletion
MSI-High	Biomarker	"High"		
CDK12	Tumor Suppressor	known or likely	contains "loss" or "deletion"	deletion
TMB-High	Biomarker	"High"		
ATM	Tumor Suppressor	known or likely	contains "loss" or "deletion"	deletion
BARD1	Tumor Suppressor	known or likely	contains "loss" or "deletion"	deletion
BRCA1	Tumor Suppressor	known or likely	contains "loss" or "deletion"	deletion
BRCA2	Tumor Suppressor	known or likely	contains "loss" or "deletion"	deletion
BRIP1	Tumor Suppressor	known or likely	contains "loss" or "deletion"	deletion
CHEK1	Tumor Suppressor	known or likely	contains "loss" or "deletion"	deletion
CHEK2	Tumor Suppressor	known or likely	contains "loss" or "deletion"	deletion
FANCA	Tumor Suppressor	known or likely	contains "loss" or "deletion"	deletion
FANCL	Tumor Suppressor	known or likely	contains "loss" or "deletion"	deletion
NBN	Tumor Suppressor	known or likely	contains "loss" or "deletion"	deletion
PALB2	Tumor Suppressor	known or likely	contains "loss" or "deletion"	deletion
RAD51	Tumor Suppressor	known or likely	contains "loss" or "deletion"	deletion
RAD51B	Tumor Suppressor	known or likely	contains "loss" or "deletion"	deletion
RAD51C	Tumor Suppressor	known or likely	contains "loss" or "deletion"	deletion
RAD51D	Tumor Suppressor	known or likely	contains "loss" or "deletion"	deletion
RAD54L	Tumor Suppressor	known or likely	contains "loss" or "deletion"	deletion
FGFR2	Oncogene	known or likely	contains "amplification"	fusion
FGFR3	Oncogene	known or likely		fusion
NTRK1	Oncogene	known or likely		fusion
NTRK2	Oncogene	known or likely		
NTRK3	Oncogene	known or likely		fusion
ABL1	Oncogene	known or likely	contains "amplification"	fusion
ALK	Oncogene	known or likely	contains "amplification"	fusion
BRAF	Oncogene	known or likely	contains "amplification"	
EGFR	Oncogene	known or likely	contains "amplification"	fusion
ERBB2	Oncogene	known or likely	contains "amplification"	
IDH1	Oncogene	known or likely		
KIT	Oncogene	known or likely	contains "amplification"	
PDGFRA	Oncogene	known or likely	contains "amplification"	fusion
RET	Oncogene	known or likely		fusion
ROS1	Oncogene	known or likely	contains "amplification"	fusion

NOTE:
SNP_Indels are found in "ShortVariants" table
CNV amplificaitons and deletions are found in "CopyNumberAlterations
Rearrangements fusions and deletions are found in "Rearrangements" table
MSI and Tumor Mutation Burden are found in "Alteration" table

eTable 3: Genes involved in studied pathways

AR Axis	Tumor Suppressors	PI3K/AKT Pathway	MMR DNA Repair Pathway	Immunotherapy Targets	HR DNA Repair Pathway	"Other" Targets	Prostate Specific PARP Inhibitor Tagets
AR	*RB1*	*PTEN*	*MLH1*	*MLH1*	*ATM*	*FGFR2*	*ATM*
SPOP	*TP53*	*AKT1*	*MSH2*	*MSH2*	*BARD1*	*FGFR3*	*BARD1*
TMPRSS2	*PTEN*	*CDKN2A*	*MSH6*	*MSH6*	*BRCA1*	*NTRK1*	*BRCA1*
		PIK3CA	*PMS2*	*PMS2*	*BRCA2*	*NTRK2*	*BRCA2*
			MSI-High	*MSI-High*	*BRIP1*	*NTRK3*	*BRIP1*
				CDK12	*CHEK1*	*ABL1*	*CHEK1*
				TMB-High	*CHEK2*	*ALK*	*CHEK2*
					FANCA	*BRAF*	*FANCL*
					FANCL	*EGFR*	*PALB2*
					NBN	*ERBB2*	*RAD51B*
					PALB2	*IDH1*	*RAD51C*
					RAD51	*KIT*	*RAD51D*
					RAD51B	*PDGFRA*	*RAD54L*
					RAD51C	*RET*	*CDK12*
					RAD51D	*ROS1*	
					RAD54L		

© 2025 Valle LF et al. *JAMA Network Open.*

eTable 3: Patient and Disease Characteristics of Self-identified non-Hispanic Black (NHB) and non-Hispanic White (NHW) Patients with Metastatic Prostate Cancer in Relation to Time of NGS Specimen Collection

Patient and Disease Characteristics Related to NGS Testing	Entire Cohort n=5015		Non-Hispanic Black n=1784		Non-Hispanic White n=3231		*p*
**Treatments started prior to NGS specimen collection**							
ADT/orchiectomy	2,225	44.0%	794	45.0%	1,431	44.0%	>0.9
ARSI	2,465	49.0%	880	49.0%	1,585	49.0%	0.9
Platinum	53	1.1%	22	1.2%	31	1.0%	0.4
Taxane	437	8.7%	168	9.4%	269	8.3%	0.2
PARP-I	7	0.1%	2	0.1%	5	0.2%	>0.9
Sipileucal-T	6	0.1%	3	0.2%	3	<0.1%	0.8
Radium	38	0.8%	6	0.3%	32	1.0%	**0.017**
**Treatments started after NGS specimen collection**							
ADT/orchiectomy	2,577	51.0%	948	53.0%	1,629	50.0%	0.069
ARSI	3,272	65.0%	1,160	65.0%	2,112	65.0%	0.8
Platinum	275	5.5%	103	5.8%	172	5.3%	0.5
Taxane	1,229	25.0%	462	26.0%	767	24.0%	0.10
PARP-I	328	6.5%	95	5.3%	233	7.2%	**0.012**
Sipileucal-T	6	0.1%	1	<0.1%	5	0.2%	0.6
Radium	45	0.9%	15	0.8%	30	0.9%	0.9
**Disease State at NGS specimen collection**							0.5
CRPC	1,545	31%	549	31%	996	31%	
CSPC	1,676	33%	579	32%	1,097	34%	
Unknown	1,794	36%	656	37%	1,138	35%	
**Age at NGS specimen collection**							**<0.001**
Mean (SD)	72.52	8.54	70.19	8.8	73.81	8.11	
Median (IQR)	73	(67,78)	70	(64,76)	74	(69,78)	
Minimum,Maximum	46	102	46	100	48	102	
**Time from Diagnosis to NGS specimen collection**							0.082
Mean (SD)	55.5	76.79	58.06	78.07	54.09	76.05	
Median (IQR)	7.84	0.00,98.41	8.52	0.00,107.15	7.67	0.00,93.36	
**Tissue Type Used for NGS**							**0.005**
Bone	176	3.5%	50	2.8%	126	3.9%	
Plasma	1,644	32.8%	629	35.3%	1,015	31.4%	
Liver	95	1.9%	37	2.1%	58	1.8%	
Lung	70	1.4%	23	1.3%	47	1.5%	
Lymph Node/Soft Tissue	670	13.4%	206	11.5%	464	14.4%	
Prostate	2,359	47.0%	838	47.0%	1,521	47.1%	
Unknown	1	0.0%	1	0.1%	0	0.0%	
**Histology of NGS specimen**	`						0.2
Adenocarcinoma	3,103	90.8%	1,081	91.4%	2,022	90.4%	
Carcinoma	237	6.9%	73	6.2%	164	7.3%	
Neuroendocrine	19	0.6%	5	0.4%	14	0.6%	
Small Cell	19	0.6%	9	0.8%	10	0.4%	
Other	41	1.2%	15	1.3%	26	1.2%	
**Tumor Purity (applies to tissue samples only)**							0.4
Mean (SD)	50.3	22.3	49.9	23.2	50.5	21.8	
Median (25%,75%)	50.1	(30.7,67.5)	50.0	(30.0,68.6)	50.3	(32.6,67.3)	
Minimum, Maximum	4.7	99.9	4.7	99.9	5.2	99.9	

© 2025 Valle LF et al. *JAMA Network Open*.

eTable 4: Oncogenic alteration rates in all tests in non-Hispanic Black (NHB) versus non-Hispanic White (NHW) patients with mPCa

Gene based analyis	Non-Hispanic White n=3231 (%)	Non-Hispanic Black n=1784 (%)	NHB-NHW (%)	Adjusted p-value
ABL1	12 (0.4%)	<11 (<0.6%)	-0.1	1
AKT1	60 (1.9%)	38 (2.1%)	0.2	0.7452
ALK	11 (0.3%)	<11 (<0.6%)	-0.1	0.6384
AR	432 (13%)	243 (14%)	1.0	0.9276
ATM	260 (8.0%)	128 (7.2%)	-0.8	0.5717
BARD1	<11 (<0.3%)	<11 (<0.6%)	0.3	0.1039
BRAF	99 (3.1%)	77 (4.3%)	1.2	0.0862
BRCA1	31 (1.0%)	14 (0.8%)	-0.2	0.8252
BRCA2	212 (6.6%)	85 (4.8%)	-1.8	**0.0441**
BRIP1	16 (0.5%)	<11 (<0.6%)	-0.2	0.6294
CDK12	144 (4.5%)	115 (6.4%)	1.9	**0.018**
CDKN2A	75 (2.3%)	18 (1.0%)	-1.3	**0.0087**
CHEK1	<11 (<0.3%)	<11 (<0.6%)	0.1	0.8282
CHEK2	169 (5.2%)	47 (2.6%)	-2.6	**0.0001**
EGFR	29 (0.9%)	20 (1.1%)	0.2	0.669
ERBB2	40 (1.2%)	17 (1.0%)	-0.2	0.6384
FANCA	19 (0.6%)	<11 (<0.6%)	-0.2	0.6384
FANCL	<11 (<0.3%)	<11 (<0.6%)	0.1	0.8252
FGFR2	<11 (<0.3%)	<11 (<0.6%)	-0.2	0.1581
FGFR3	<11 (<0.3%)	<11 (<0.6%)	0.2	0.1581
IDH1	13 (0.4%)	<11 (<0.6%)	0.0	1
KIT	<11 (<0.3%)	<11 (<0.6%)	0.2	0.3884
MLH1	11 (0.3%)	<11 (<0.6%)	0.0	1
MSH2	33 (1.0%)	33 (1.8%)	0.8	0.0747
MSH6	37 (1.1%)	23 (1.3%)	0.2	0.8252
NBN	12 (0.4%)	12 (0.7%)	0.3	0.332
NTRK1	<11 (<0.3%)	<11 (<0.6%)	0.0	1
NTRK2	<11 (<0.3%)	<11 (<0.6%)	0.0	0.7698
NTRK3	<11 (<0.3%)	<11 (<0.6%)	-0.1	0.5717
PALB2	22 (0.7%)	<11 (<0.6%)	-0.3	0.6237
PDGFRA	<11 (<0.3%)	<11 (<0.6%)	0.3	0.8951
PIK3CA	209 (6.5%)	80 (4.5%)	-2.0	**0.0187**
PMS2	11 (0.3%)	19 (1.1%)	0.8	**0.0187**
PTEN	747 (23%)	254 (14%)	-9.0	**0**
RAD51	<11 (<0.3%)	<11 (<0.6%)	0.2	0.3884
RAD51B	<11 (<0.3%)	<11 (<0.6%)	0.2	0.8252
RAD51C	<11 (<0.3%)	<11 (<0.6%)	0.2	0.5215
RAD51D	<11 (<0.3%)	<11 (<0.6%)	-0.1	0.8252
RAD54L	<11 (<0.3%)	<11 (<0.6%)	0.2	0.6384
RB1	120 (3.7%)	50 (2.8%)	-0.9	0.2675
RET	<11 (<0.3%)	13 (0.7%)	0.4	0.0862
ROS1	<11 (<0.3%)	<11 (<0.6%)	0.0	1
SPOP	287 (8.9%)	210 (12%)	3.1	**0.0103**
TMPRSS2	893 (28%)	225 (13%)	-15.0	**0**
TP53	1,223 (38%)	519 (29%)	-9.0	**0**

Pathway-based analysis	Non-Hispanic White n=3231 (%)	Non-Hispanic Black n=1784 (%)	% B - W	Adjusted p-value
AKT/PI3K Pathway	965 (30%)	355 (20%)	-10.0	**0**
AR Axis	1,429 (44%)	616 (35%)	-9.0	**0**
DNA Repair Genes	709 (22%)	304 (17%)	-5.0	**0.0001**
PCa-Specific PARPi Targets	802 (25%)	394 (22%)	-3.0	**0.0335**
Immunotherapy Targets	232 (7.2%)	195 (11%)	3.8	**0**
Mismatch Repair Deficiency	96 (3.0%)	82 (4.6%)	1.6	**0.0053**
Other targeted therapy	236 (7.3%)	155 (8.7%)	1.4	0.088
Tumor suppressor genes	1,665 (52%)	682 (38%)	-14.0	**0**

Microsatellite Status	Non-Hispanic White n=1991 (%)	Non-Hispanic Black n=1032 (%)	% B - W	Adjusted p-value
MSI-High Status	53 (2.7%)	41 (4.7%)	2.0	**0.029**

TMB status	Non-Hispanic White n=3057 (%)	Non-Hispanic Black n=1683 (%)	% B - W	Adjusted p-value
# with TMB-High Status	47 (1.5%)	37 (2.1%)	0.6	0.2675

(1) Rest of genes have <11 samples with alterations

© 2025 Valle LF et al. *JAMA Network Open.*

eTable 5: Oncogenic alteration rates in primary prostate versus metastasis and liquid biopsies in patients with mPCa

Gene-Based analysis	Primary n=2539 (%)	Metastasis n=1011 (%)	Adjusted p-value Metastasis vs Primary	Liquid n=1644 (%)	Adjusted p-value Liquid vs Primary
ABL1	<11 (<0.4%)	<11 (<1%)	0.4999	<11 (<0.7%)	0.7681
AKT1	44 (1.9%)	36 (3.6%)	**0.0336**	18 (1.1%)	0.1767
ALK	<11 (<0.4%)	<11 (<1%)	0.5831	<11 (<0.7%)	0.9214
AR	37 (1.6%)	225 (22%)	**0**	413 (25%)	**0**
ATM	110 (4.7%)	58 (5.7%)	0.4999	220 (13%)	**0**
BARD1	<11 (<0.4%)	<11 (<1%)	0.5624	<11 (<0.7%)	0.9197
BRAF	91 (3.9%)	41 (4.1%)	0.9549	44 (2.7%)	0.1379
BRCA1	18 (0.8%)	11 (1.1%)	0.7227	16 (1.0%)	0.7402
BRCA2	135 (5.7%)	74 (7.3%)	0.2703	88 (5.4%)	0.8157
BRIP1	<11 (<0.4%)	<11 (<1%)	1	12 (0.7%)	0.0925
CDK12	139 (5.9%)	64 (6.3%)	0.9111	56 (3.4%)	**0.0013**
CDKN2A	48 (2.0%)	34 (3.4%)	0.1438	11 (0.7%)	**0.0013**
CHEK1	<11 (<0.4%)	<11 (<1%)	1	<11 (<0.7%)	0.462
CHEK2	30 (1.3%)	18 (1.8%)	0.5686	168 (10%)	**0**
EGFR	13 (0.6%)	16 (1.6%)	**0.045**	20 (1.2%)	0.0925
ERBB2	29 (1.2%)	22 (2.2%)	0.2126	<11 (<0.7%)	**0.0113**
FANCA	15 (0.6%)	<11 (<1%)	0.9735	<11 (<0.7%)	0.4958
FANCL	<11 (<0.4%)	<11 (<1%)	1	<11 (<0.7%)	1
FGFR2	<11 (<0.4%)	<11 (<1%)	0.2703	<11 (<0.7%)	0.3722
FGFR3	<11 (<0.4%)	<11 (<1%)	0.5831	<11 (<0.7%)	0.0925
IDH1	<11 (<0.4%)	<11 (<1%)	0.5831	<11 (<0.7%)	0.3831
KIT	<11 (<0.4%)	<11 (<1%)	0.9308	<11 (<0.7%)	1
MLH1	<11 (<0.4%)	<11 (<1%)	1	<11 (<0.7%)	0.4911
MSH2	26 (1.1%)	27 (2.7%)	**0.0126**	13 (0.8%)	0.6483
MSH6	29 (1.2%)	15 (1.5%)	0.9111	16 (1.0%)	0.7681
NBN	<11 (<0.4%)	<11 (<1%)	0.4865	<11 (<0.7%)	0.54
NTRK1	<11 (<0.4%)	<11 (<1%)	1	<11 (<0.7%)	1
NTRK2	<11 (<0.4%)	<11 (<1%)	1	<11 (<0.7%)	1
NTRK3	<11 (<0.4%)	<11 (<1%)	0.8562	<11 (<0.7%)	0.7681
PALB2	17 (0.7%)	<11 (<1%)	0.2703	11 (0.7%)	1
PDGFRA	<11 (<0.4%)	<11 (<1%)	0.9308	<11 (<0.7%)	0.7681
PIK3CA	157 (6.7%)	71 (7.0%)	0.9308	60 (3.6%)	**0.0001**
PMS2	15 (0.6%)	10 (1.0%)	0.5686	<11 (<0.7%)	0.3728
PTEN	503 (21%)	311 (31%)	**0**	186 (11%)	**0**
RAD51	<11 (<0.4%)	<11 (<1%)	0.9111	<11 (<0.7%)	0.4976
RAD51B	<11 (<0.4%)	<11 (<1%)	0.9308	<11 (<0.7%)	0.2043
RAD51C	<11 (<0.4%)	<11 (<1%)	0.9111	<11 (<0.7%)	1
RAD51D	<11 (<0.4%)	<11 (<1%)	0.2703	<11 (<0.7%)	1
RAD54L	<11 (<0.4%)	<11 (<1%)	1	<11 (<0.7%)	0.3722
RB1	45 (1.9%)	62 (6.1%)	**0**	63 (3.8%)	**0.0013**
RET	<11 (<0.4%)	<11 (<1%)	0.8873	<11 (<0.7%)	0.54
ROS1	<11 (<0.4%)	<11 (<1%)	1	<11 (<0.7%)	1
SPOP	301 (13%)	112 (11%)	0.4999	84 (5.1%)	**0**
TMPRSS2	688 (29%)	274 (27%)	0.5353	156 (9.5%)	**0**
TP53	708 (30%)	376 (37%)	**0.0007**	657 (40%)	**0**

© 2025 Valle LF et al. *JAMA Network Open.*

Pathway-based analysis	Primary n=2539 (%)	Metastasis n=1011 (%)	Adjusted p-value Metastasis vs Primary	Liquid n=1644 (%)	Adjusted p-value Liquid vs Primary
AKT/PI3K Pathway	687 (29%)	383 (38%)	**0**	249 (15%)	**0**
AR Axis	1,002 (42%)	514 (51%)	**0**	529 (32%)	**0**
DNA Repair Genes	344 (15%)	184 (18%)	**0.011**	485 (30%)	**0**
PCar-Specific PARPi Targets	447 (19%)	235 (23%)	**0.008**	514 (31%)	**0**
Immunotherapy Targets	212 (9.0%)	117 (12%)	**0.0226**	98 (6.0%)	**0.0006**
Mismatch Repair Deficiency	80 (3.4%)	54 (5.3%)	**0.011**	44 (2.7%)	0.304
Other targeted therapy	173 (7.3%)	105 (10%)	**0.008**	113 (6.9%)	0.6179
Tumor suppressor genes	1,048 (44%)	553 (55%)	**0**	745 (45%)	0.583008

Microsatellite Status	Primary n=1988 (%)	Metastasis n=932 (%)	Adjusted p-value Metastasis vs Primary	Liquid n=102 (%)	Adjusted p-value Liquid vs Primary
MSI-High Status	42 (2.3%)	31 (3.5%)	0.2703	21 (21%)	**0**

TMB status	Primary n=2133 (%)	Metastasis n=970 (%)	Adjusted p-value Metastasis vs Primary	Liquid n=1636 (%)	Adjusted p-value Liquid vs Primary
TMB-High Status	33 (1.4%)	27 (2.7%)	0.0877	24 (1.5%)	1

(1) Rest of genes have <11 samples with alterations

© 2025 Valle LF et al. *JAMA Network Open*.

eTable 6: Oncogenic alteration rates in non-Hispanic Black (NHB) versus non-Hispanic White (NHW) patients with mPCa stratified by tissue type tested

Gene-Based analysis	Non-Hispanic Black, Primary, n=838	Non-Hispanic White, Primary, n=1,521	Adj. p-value	Non-Hispanic Black, Metastasis, n=316	Non-Hispanic White, Metastasis, n=695	Adj. p-value	Non-Hispanic Black, Plasma, n=629	Non-Hispanic White, Plasma, n=1,015	Adj. p-value
AR	14 (1.7%)	23 (1.5%)	1	68 (22%)	157 (23%)	1	161 (26%)	252 (25%)	1
BRCA2	36 (4.3%)	99 (6.5%)	0.1007	18 (5.7%)	56 (8.1%)	0.8334	31 (4.9%)	57 (5.6%)	0.9318
CDK12	67 (8.0%)	72 (4.7%)	**0.0121**	23 (7.3%)	41 (5.9%)	1	25 (4.0%)	31 (3.1%)	0.8769
CDKN2A	12 (1.4%)	36 (2.4%)	0.3858	<11 (<3.5%)	28 (4.0%)	0.5696	<11 (<1.7%)	11 (1.1%)	0.0839
CHEK2	<11 (<1.3%)	27 (1.8%)	**0.0157**	<11 (<3.5%)	16 (2.3%)	0.5696	42 (6.7%)	126 (12%)	**0.0036**
PIK3CA	39 (4.7%)	118 (7.8%)	**0.0157**	20 (6.3%)	51 (7.3%)	1	20 (3.2%)	40 (3.9%)	0.8851
PMS2	13 (1.6%)	<11 (<0.7%)	**0.0006**	<11 (<3.5%)	<11 (<1.6%)	1	<11 (<1.7%)	<11 (<1.1%)	1
PTEN	113 (13%)	390 (26%)	**0**	69 (22%)	242 (35%)	**0.0006**	71 (11%)	115 (11%)	1
SPOP	138 (16%)	163 (11%)	**0.0006**	34 (11%)	78 (11%)	1	38 (6.0%)	46 (4.5%)	0.8769
TMPRSS2	146 (17%)	542 (36%)	**0**	45 (14%)	229 (33%)	**0**	34 (5.4%)	122 (12%)	**0.0003**
TP53	205 (24%)	503 (33%)	**0.0002**	97 (31%)	279 (40%)	0.0629	216 (34%)	441 (43%)	**0.0044**

Pathway-based analysis	Non-Hispanic Black, Primary, n=838	Non-Hispanic White, Primary, n=1,521	Adjusted p-value	Non-Hispanic Black, Metastasis, n=316	Non-Hispanic White, Metastasis, n=695	Adjusted p-value	Non-Hispanic Black, Plasma, n=629	Non-Hispanic White, Plasma, n=1,015	Adjusted p-value
AKT/PI3K Pathway	171 (20%)	516 (34%)	**0**	94 (30%)	289 (42%)	**0.0009**	89 (14%)	160 (16%)	0.5961
AR Axis	295 (35%)	707 (46%)	**0**	126 (40%)	388 (56%)	**0**	195 (31%)	334 (33%)	0.5961
DNA Repair Genes	95 (11%)	249 (16%)	**0.0011**	54 (17%)	130 (19%)	0.6833	155 (25%)	330 (33%)	**0.0055**
PCa-Specific PARPi Targets	151 (18%)	296 (19%)	0.4106	72 (23%)	163 (23%)	0.872443	171 (27%)	343 (34%)	**0.014**
Immunotherapy Targets	109 (13%)	103 (6.8%)	**0**	40 (13%)	77 (11%)	0.6833	46 (7.3%)	52 (5.1%)	0.1717
Mismatch Repair Deficiency	44 (5.3%)	36 (2.4%)	**0.0004**	19 (6.0%)	35 (5.0%)	0.6833	19 (3.0%)	25 (2.5%)	0.6067
Other targeted therapy	84 (10%)	89 (5.9%)	**0.0004**	30 (9.5%)	75 (11%)	0.6833	41 (6.5%)	72 (7.1%)	0.6892
Tumor suppressor genes	284 (34%)	764 (50%)	**0**	142 (45%)	411 (59%)	**0.0001**	255 (41%)	490 (48%)	**0.0089**

Microsatellite Status	Non-Hispanic Black, Primary, n=696	Non-Hispanic White, Primary, n=1,292	Adjusted p-value	Non-Hispanic Black, Metastasis, n=292	Non-Hispanic White, Metastasis, n=640	Adjusted p-value	Non-Hispanic Black, Plasma,	Non-Hispanic White, Plasma, n=59	Adjusted p-value
MSI-High Status	20 (3.5%)	22 (1.7%)	0.1007	13 (5.0%)	18 (2.8%)	0.5696	<11 (<25.6%	13 (22%)	1

TMB status	Non-Hispanic Black, Primary, n=748	Non-Hispanic White, Primary, n=1,385	Adjusted p-value	Non-Hispanic Black, Metastasis, n=305	Non-Hispanic White, Metastasis, n=665	Adjusted p-value	Non-Hispanic Black, Plasma, n=629	Non-Hispanic White, Plasma, n=1,007	Adjusted p-value
TMB-High Status	16 (1.9%)	17 (1.1%)	0.3929	9 (2.8%)	18 (2.6%)	1	12 (1.9%)	12 (1.2%)	0.8769

© 2025 Valle LF et al. *JAMA Network Open.*

eTable 7: Association of race with alteration frequency in metastatic prostate cancer

	All Samples(1)		Primary Tissues(2)		Metastatic Tissues(2)		Plasma(2)	
**Gene or Pathway**	**OR (95% CI)**	**p-value**	**OR (95% CI)**	**p-value**	**OR (95% CI)**	**p-value**	**OR (95% CI)**	**p-value**
AR Axis	0.7 (0.5 , 0.9)	**0.0012**	0.5 (0.3 , 0.7)	**< 0.0001**	0.6 (0.3 , 1.1)	0.0721	1.2 (0.8 , 1.8)	0.423
TMPRSS2	0.3 (0.2 , 0.4)	**< 0.0001**	0.2 (0.1 , 0.3)	**< 0.0001**	0.1 (0.1 , 0.3)	**< 0.0001**	0.5 (0.2 , 1.1)	0.101
SPOP	1.7 (1.2 , 2.6)	**0.0059**	1.7 (1.0 , 2.8)	**0.034**	1.3 (0.4 , 3.5)	0.6673	3.4 (1.2 , 9.5)	**0.0183**
Tumor Suppressor Genes	0.7 (0.5 , 0.8)	**4e-04**	0.5 (0.4 , 0.7)	**2e-04**	0.6 (0.3 , 1.0)	0.0712	1.0 (0.7 , 1.4)	0.8225
TP53	0.7 (0.6 , 0.9)	**0.0132**	0.8 (0.5 , 1.1)	0.2182	0.3 (0.2 , 0.7)	**0.0029**	0.9 (0.6 , 1.3)	0.5102
PTEN	0.5 (0.4 , 0.7)	**< 0.0001**	0.4 (0.2 , 0.6)	**< 0.0001**	0.6 (0.3 , 1.2)	0.1759	0.9 (0.5 , 1.7)	0.7745
Immunotherapy Targets	1.7 (1.1 , 2.5)	**0.018**	2.1 (1.2 , 3.9)	**0.0119**	1.4 (0.5 , 3.7)	0.4512	0.9 (0.3 , 2.6)	0.9158
MSI High Status	3.1 (1.1 , 9.4)	**0.0348**	3.5 (0.6 , 21.8)	0.1507	1.3 (0.1 , 16.5)	0.8524	Not Reportable	N/A
Mismatch Repair Pathway	1.8 (1.0 , 3.4)	0.063	3.9 (1.4 , 12.3)	**0.0144**	0.9 (0.2 , 3.9)	0.9372	1.2 (0.3 , 4.4)	0.8264
CDK12	1.6 (0.9 , 2.7)	0.0891	Not Reportable	N/A	2.1 (0.6 , 7.2)	0.2505	0.4 (0.1 , 1.8)	0.2594
PMS2	8.6 (1.0 , 133.5)	0.0725	Not Reportable	N/A	Not Reportable	N/A	Not Reportable	N/A
AKT/PI3K Pathway	0.6 (0.4 , 0.7)	**< 0.0001**	0.5 (0.3 , 0.7)	**1e-04**	0.7 (0.4 , 1.3)	0.2199	0.7 (0.4 , 1.3)	0.2979
CDKN2A	0.7 (0.2 , 1.7)	0.4421	1.2 (0.3 , 4.5)	0.7928	0.5 (0.1 , 3.0)	0.5219	Not Reportable	N/A
PIK3CA	0.7 (0.4 , 1.2)	0.2087	0.7 (0.3 , 1.4)	0.2751	0.6 (0.2 , 1.9)	0.4502	0.6 (0.2 , 1.9)	0.4236
DNA Repair Genes	0.7 (0.5 , 0.9)	**0.0069**	0.5 (0.3 , 0.9)	**0.0215**	0.5 (0.2 , 1.1)	0.0963	0.8 (0.5 , 1.2)	0.3375
PCa-Specific PARPi Targets	0.8 (0.6 , 1.1)	0.2098	0.8 (0.5 , 1.3)	0.4507	0.8 (0.4 , 1.7)	0.586	0.8 (0.5 , 1.2)	0.2992
BRCA2	0.8 (0.5 , 1.2)	0.2755	1.1 (0.5 , 2.4)	0.8471	0.3 (0.1 , 1.1)	0.1079	0.8 (0.3 , 1.8)	0.549
CHEK2	0.5 (0.3 , 1.0)	**0.0446**	0.4 (0.0 , 2.0)	0.2957	37.6 (0 , NA)	0.5995	Not Reportable	N/A

(1) Multivariable logistic regression analysis adjusted for sample type, age at diagnosis, age at sample collection, de novo metastatic status, disease state, PSA at diagnosis, Gleason grade, military exposures, pathological diagnosis, CCI, ADI, smoking and marital status

(2) Multivariable logistic regression analysis adjusted for age of dx, age of sample collection, de novo metastatic status, disease state, PSA at dx, grade,military exposures, pathological diagnosis, CCI, ADI, smoking and marital status

Reference is NHW patients

© 2025 Valle LF et al. *JAMA Network Open.*

eTable 8: Association of overall survival with oncogenic alterations in individual genes and pathways

	Non-Hispanic White		Non-Hispanic Black	
**Gene or Pathway**	**HR (95% CI)**	**p-value**	**HR (95% CI)**	**p-value**
AR Axis	1.28 (1.05, 1.56)	**0.01566**	1.33 (0.96, 1.85)	0.08246
TMPRSS2	1.06 (0.86, 1.31)	0.59685	0.87 (0.51, 1.47)	0.59809
SPOP	0.80 (0.54, 1.18)	0.25717	0.94 (0.53, 1.66)	0.82978
Tumor Suppressor Genes	1.52 (1.25, 1.85)	**< 0.001**	1.54 (1.13, 2.11)	**0.00645**
TP53	1.61 (1.33, 1.95)	**< 0.001**	2.03 (1.46, 2.82)	**< 0.001**
PTEN	1.15 (0.93, 1.43)	0.20399	1.14 (0.74, 1.77)	0.5443
Immunotherapy Targets	1.44 (1.02, 2.02)	**0.03694**	1.53 (0.91, 2.59)	0.10842
MSI High Status	3.55 (1.67, 7.55)	**< 0.001**	2.26 (0.70, 7.30)	0.17247
Mismatch Repair Deficiency	2.15 (1.31, 3.53)	**0.00260**	1.12 (0.53, 2.37)	0.76187
CDK12	1.08 (0.69, 1.68)	0.73917	2.04 (1.13, 3.67)	**0.01801**
PMS2	0.82 (0.11, 6.35)	0.8532	Not Reportable	N/A
AKT/PI3K Pathway	1.13 (0.92, 1.39)	0.24568	1.02 (0.68, 1.54)	0.90676
CDKN2A	0.71 (0.35, 1.42)	0.33415	0.97 (0.28, 3.38)	0.96077
PIK3CA	1.20 (0.84, 1.70)	0.32234	1.03 (0.51, 2.09)	0.9289
DNA Repair Genes	1.04 (0.82, 1.31)	0.75909	0.88 (0.57, 1.36)	0.55366
PCa-Specific PARPi Targets	1.05 (0.85, 1.31)	0.63386	1.17 (0.80, 1.71)	0.43277
BRCA2	1.22 (0.87, 1.71)	0.24309	1.81 (0.98, 3.34)	0.05847
CHEK2	0.82 (0.50, 1.33)	0.41467	0.50 (0.19, 1.34)	0.17059

© 2025 Valle LF et al. *JAMA Network Open*.
