# Supplementary material for: Somatic Tumor Next-Generation Sequencing in US Veterans With Metastatic Prostate Cancer
Source: JAMA Netw Open. 2025 May 12;8(5):e259119. doi: 10.1001/jamanetworkopen.2025.9119 (PMC12070238; doi:10.1001/jamanetworkopen.2025.9119)
Supplement: Supplement 2. — Data Sharing Statement [file jamanetwopen-e259119-s002.pdf]

Data Sharing Statement

Valle. Somatic Tumor Next-Generation Sequencing in US Veterans With Metastatic Prostate Cancer. *JAMA Netw Open*. Published May 08, 2025. doi:10.1001/jamanetworkopen.2025.9119

Data
Data available: No

Additional Information
Explanation for why data not available: Individual Veteran data is not shareable per VA policy
